# Multi-integrated approach for unraveling small open reading frames potentially associated with secondary metabolism in *Streptomyces*

**DOI:** 10.1128/msystems.00245-23

**Published:** 2023-09-15

**Authors:** Si-Min Fan, Ze-Qi Li, Shi-Zhe Zhang, Liang-Yu Chen, Xi-Ying Wei, Jian Liang, Xin-Qing Zhao, Chun Su

**Affiliations:** 1 National Engineering Laboratory for Resource Developing of Endangered Chinese Crude Drugs in Northwest China, College of Life Sciences, Shaanxi Normal University, Shaanxi, China; 2 ProteinT (Tianjin) biotechnology Co. Ltd., Tianjin, China; 3 College of Biology and Geography, Yili Normal University, Yining, China; 4 State Key Laboratory of Microbial Metabolism, School of Life Sciences and Biotechnology, Shanghai Jiao Tong University, Shanghai Jiao, China; Ocean University of China, Qingdao, Shandong Province, China

**Keywords:** smORF-encoded peptides, peptidogenomics, *de novo *sequencing, secondary metabolism, *Streptomyces*

## Abstract

Small open reading frames (smORFs) are widely distributed in various living organisms. However, their functions remain largely unexplored. In addition, annotation and detection of smORFs are limited using existing methods and hindered by their specific properties. In this study, we systematically investigated smORFs and smORF-encoded peptides (SEPs) in *Streptomyces*, which are well-known bacterial producers of diverse bioactive secondary metabolites. We established a peptidogenomic workflow based on multi-integrated comprehensive database search and database-independent *de novo* sequencing to identify smORFs in *Streptomyces xinghaiensis* NRRL B-24674^T^ (S187). In addition, we described SEPome related to the secondary metabolism, which include 68 novel SEPs and 79 common smORFs with *Streptomyces coelicolor* A3 (2). Functional analysis of universal smORFs revealed enrichment in biosynthetic processes, stress response, ribosomes, and nucleic acid binding. Meanwhile, 5 Cryptic smORF-encoded Peptides (CSEPs) distributed in non-annotated regions of the genome, and non-coding RNAs could encode for CSEPs. A total of 66 new RNAs, including 32 non-coding RNAs (ncRNAs) were revealed, and 4 ncRNA-encoded peptides were identified. Furthermore, an investigation of carbon metabolism showed that NagE functions in spore formation and secondary metabolism in *Streptomyces*. Particularly, NagE was observed to function in the biosynthesis of anti-complement agents in *S. xinghaiensis,* suggesting a novel role of the phosphoenolpyruvate phosphotransferase system in microbial secondary metabolism. We thus provide an effective strategy for analyzing public data sets of model strains to identify smORFs for non-model species. The ncRNAs and SEPs present rich sources for engineering streptomycetes to produce bioactive compounds.

**Importance:**

Due to their small size and special chemical features, small open reading frame (smORF)-encoding peptides (SEPs) are often neglected. However, they may play critical roles in regulating gene expression, enzyme activity, and metabolite production. Studies on bacterial microproteins have mainly focused on pathogenic bacteria, which are importance to systematically investigate SEPs in streptomycetes and are rich sources of bioactive secondary metabolites. Our study is the first to perform a global identification of smORFs in streptomycetes. We established a peptidogenomic workflow for non-model microbial strains and identified multiple novel smORFs that are potentially linked to secondary metabolism in streptomycetes. Our multi-integrated approach in this study is meaningful to improve the quality and quantity of the detected smORFs. Ultimately, the workflow we established could be extended to other organisms and would benefit the genome mining of microproteins with critical functions for regulation and engineering useful microorganisms.

## INTRODUCTION

Small open reading frames (smORFs) are DNA sequences that can be translated from less than 100 codons in eukaryotes and bacteria (1). They are widely distributed in the genomes of various species. However, smORFs have typically been deemed non-coding based on their length, and a few contain non-AUG start codons (2). Classical algorithms neglect smORFs in evaluating protein-coding capacity (3). In previous bioinformatic analyses, these transcripts were usually annotated as non-coding RNA (ncRNA) or junk proteins (4). Nevertheless, these long non-coding RNAs (lncRNAs), circular RNAs (circRNAs) in eukaryotes, and ncRNA in prokaryotes can be translated into small proteins with advances in high-throughput sequencing, ribosome footprinting, and proteomics (5 – 10). Moreover, recent evidence suggests that processed and modified smORF-encoded peptides (SEPs) are instrumental in the physiological and pathophysiological functions of eukaryotes (11 – 14). SEPs play vital regulatory roles in prokaryotes concerning cellular stress, membrane transport, and antibiotic biosynthesis (15, 16). Most current work on smORFs and SEPs in bacteria mainly focuses on the virulence and stress response of pathogenic bacteria (17 – 20). However, SEPs related to the regulation of metabolites production in beneficial bacteria have been rarely been studied (16, 21). Therefore, systematic exploration of smORFs in bacteria with potential for industrial applications is urgently needed.

Actinomycetes are a rich source of structurally diverse bioactive secondary metabolites that receive constant attention for drug discovery. Due to their unique living environment, marine streptomycetes often encounter various external pressures. They are important players in producing unique bioactive secondary metabolites (22, 23). Considering that SEPs often have vital effects in allowing living organisms to accommodate multiple environmental conditions, exploring microproteins from marine actinomycetes is of great interest (24). However, the functions and properties of microproteins in most *Streptomyces* species have not yet been studied. Studies on mining smORFs from *Streptomyces* will likely to reveal their roles in secondary metabolism and elucidate influencing factors and regulatory mechanisms in the biosynthesis of useful secondary metabolites.

In recent years, various bioinformatic-based methods have been developed to explore thousands of smORFs and ncRNAs with advances in next-generation sequencing (NGS) technology (15, 25, 26). A multi-integrated approach can combine different databases, such as genomic and transcriptomic databases, to improve prediction accuracy, and has attracted increasing attention for SEP discovery (27, 28). Peptidogenomics is derived by combining genome mining and peptidomics and can be beneficial in identifying complete ORFs and hidden SEPs (29). Database (DB) search has been the main method of analysis for peptidomics because of their high accuracy and simple operation (30 – 32). Completely annotated protein databases are available for humans, mice, and common laboratory model organisms. However, it is difficult to identify novel SEPs in non-model organisms because of a lack of available public databases. *De novo* sequencing, which involves directly inferring peptide sequences by comparing mass differences from MS spectra to amino acid residues, avoids dependence on databases to discover more novel SEPs (33, 34). Given the distinct advantages of DB search and *de novo* sequencing, combining these two approaches seems conducive to identifying the SEPs of non-model organisms.

Non-model industrial microorganisms exhibit unique and diverse metabolic characteristics, offering the potential for in-depth investigations of secondary metabolic pathways. *Streptomyces xinghaiensis*, a marine sediment-derived streptomycete isolated by our group, is characterized as a novel species and was named *S. xinghaiensis* NRRL B-24674^T^ (referred to as strain S187) (35). Various strains of *S. xinghaiensis* have been identified using genomic and interspecific analyses compared to S187 (36 – 38). *S. xinghaiensis* S187 has been revealed to have significant applications potential for mining new compounds and biosynthetic enzymes (39, 40). Importantly, S187 metabolites also exhibited strong anti-complement activity and might potentially be used as a source of novel microbially derived agents for developing autoimmune disease drugs (41). Our genome mining analysis of *S. xinghaiensis* identified a potential glycopeptide (named as xinghaimycin) biosynthesis gene cluster (BGC), which showed 93% overall similarity with that of the known anticomplement agent complestatin (42). Although the structure of xinghaimycin remains unsolved due to low concentration in the fermentation broth, further analysis revealed the potential products of the xinghaimycin BGC are related to anticomplement activity and antibacterial activity (43).

To improve the production of the anti-complement active compounds and investigate the biosynthetic mechanism of S187, it is important to understand the role of SEPs in regulating secondary metabolism in *Streptomyces*. In this study, we proposed an optimized peptidogenomic workflow, including sample preparation, comprehensive database construction, and high-precision DIA for mass spectrometric detection. Simultaneously, a combination of DB search and database-independent *de novo* sequencing was used to identify as many novel SEPs as possible. To the best of our knowledge, this is the first report to combine peptidogenomics with *de novo* sequencing to identify SEPs related to secondary metabolism. Our results provide a basis for studies on SEPs in *Streptomyces* and other living organisms.

## MATERIALS AND METHODS

### Bacterial strains and culture conditions

The strains and plasmids used in this study are listed in Table S1. Plasmids were propagated in *Escherichia coli* DH5α cells cultured in Luria-Bertani broth with 50 µg/mL apramycin at 37°C. MS agar medium (2.0% soy flour, 2.0% mannitol, 2.0% agar, 10 mM MgCl_2_) was used for intergeneric conjugation between *E. coli* ET12567/pUZ8002 and streptomycete. For spore preparation, S187, *Streptomyces coelicolor* M145 (*S. coelicolor* A3 (2) without the endogenous plasmid), and their derivatives were maintained on the MS medium. For seed cultures, S187 and *S. coelicolor* M145 were cultivated in TSB medium (1.7% tryptone, 3% soy peptone, 0.5% glucose, 0.5% NaCl, 0.25% K_2_HPO_4_, unadjusted pH) at 28°C and shaken at 200 rpm for 36 hours. For fermentation and analysis of the metabolites, S187 was cultivated in an M33 medium (3.0% soluble starch, 1.0% soy flour, 0.25% yeast extract, 0.3% CaCO_3_, pH 7.2) for 48 and 120 hours. For growth and fermentation curve measurements, 1 mL (10^8^ CFU/mL) of spore suspension was sampled every 4 or 12 hours.

### Sample preparation for peptidogenomic analysis

Samples taken at different time points (36, 48, 72, and 120 hours) were rapidly frozen in liquid nitrogen and ground into powder. Endogenous peptides were extracted using 3:1:4 (vol/vol) methanol/chloroform/water, and the aqueous supernatant was passed through a 10 kDa protein ultrafiltration membrane to enrich SEPs. Certain peptides from different samples were mixed in equal volumes. The mixed sample (mix-sample) and the remaining peptides (single-sample) were desalted using a C18 cartridge to remove urea. Endogenous peptides were loaded onto a C18 tip and collected in three fractions. All fractions were dried under vacuum and reconstituted in water containing 0.1% (vol/vol) formic acid (FA). A standard (0.2 µL) was added to the fractionated samples before subsequent analyses.

### DDA and DIA liquid chromatography-tandem mass spectrometry

DDA and DIA mass spectrometry data were obtained using an Orbitrap Q-Exactive HF mass spectrometer (Thermo Fisher Scientific, Bremen, Germany) coupled with an online Easy-nLC 1200 nano-high performance liquid chromatography (HPLC) system (Thermo Fisher Scientific, Bremen, Germany). For transition library construction, a sample containing 1 µg of the total peptide from a fractionated sample reconstituted in 0.1% FA was injected onto a homemade C18 Nano-Trap column (2 cm × 100 µm, 3 µm, Thermo Fisher Scientific, Bremen, Germany). Peptides were separated on an analytical column (25 cm × 5 µm, 100 A) using a 90 minutes linear gradient of 0 to 100% of eluent B (0.1% FA in 80% acetonitrile [ACN], 20% water) in eluent A (0.1% FA in water) at a flow rate of 600 nL/minute. The detailed solvent gradient was as follows: 6%–12% B, 8 minutes; 12%–30% B, 55 minutes; 30%–40% B, 12 minutes; and 40%–95% B, 15 minutes. The Orbitrap Fusion mass spectrometer was operated in DDA mode using the Xcalibur 3.0 software with a 1.8 kV electrospray voltage. The full scan was processed in Orbitrap from m/z 350 to 1,200, followed by data-dependent MS_2_ scans in an ion-routing multipole at 30% normalized collision energy (HCD). The resolution was 60,000 for the full scan mode and 15,000 for the MS_2_ mode. The maximum scan time was 50 ms for full scans and 22 ms for MS_2_ scans. MS_1_ resolution was set to 120,000 and MS_2_ resolution to 30,000. The m/z range was 350–1,500. The DIA settings used a normalized collision energy of 33%.

### smORF database construction

To generate a comprehensive S187 smORF database and assess the characteristics of smORFs, we chose all putative ORFs with a size ≤300 bases from the S187 genome six-frame translation database and used alternative start and stop codons (start: ATG, GTG, CTG, and TTG; stop: TAG, TAA, and TGA) in the online web resource OrfFinder (43). To obtain the S187 smORF database with higher confidence, the Prodigal prokaryotic dynamic programming genetic algorithm v2.6.3 (44) was used to re-predict the full ORF set for S187, obtaining information on each potential start site and including parameters such as confidence scores and ribosome binding site motifs. Alternative start codons for bacteria were chosen, and ORFs with confidence levels of ≥90 and encoded amino acid lengths of ≤100 aa were selected as a library of SEPs with high coding potential.

### Small RNAs and predicted SEP database construction

Seven sets of wild-type *S. coelicolor* A3 (2) raw transcriptome data were selected from available NCBI online SRA databases. Transcriptome analysis was carried out on the following data sets: SRR13349472, SRR13349473, SRR10011614, SRR10011615, SRR5371191, SRR5371192, and SRR5371193 (45 – 47). RNA-seq raw data were processed through a quality check using FastQC, and Trimmomatic (48) was used to remove adapters and low-quality sequences. Bowtie2 (49) and STAR (50) were simultaneously used to map sequences to the reference genome of *S. coelicolor* A3 (2) to ensure the accuracy of the results, and SAMtools was used to generate binary sequencing files (*.bam). RSEM and featureCounts (51) were used for quantification. The transcript files were filtered under FPKM > 1 and nucleotide length ≤300 bases to generate a small RNA (smRNA) database of the model strains. Putative smRNAs were translated using EMBOSS Transeq. The SEP database of the model strain *S. coelicolor* A3 (2) was compared to the S187 genome using tBLASTn or BLASTp to construct the predicted SEP database.

### RNA-seq data analysis

mRNAs with polyA structure were enriched from total RNA using oligo(dT) magnetic beads, and ion interruption was used to break the RNA into fragments of approximately 300 bp. Using RNA as the template, library fragments were enriched using PCR amplification, followed by library selection based on a fragment size of 450 bp. After RNA extraction, purification, and library construction, libraries were sequenced using NGS based on the Illumina HiSeq sequencing platform with paired-end sequencing. Quality control, read mapping, and quantification of transcriptome data from S187 were performed using Trimmomatic, Bowtie2, and featureCounts, respectively. The transcript file was filtered with FPKM >1 and nucleotide length ≤300 bases to generate an SEP database based on the S187 transcriptome. The database was merged with the predicted SEP database to construct a *Streptomyces* SEP database for spectral matching and DB searches.

### Database search of MS data

Transcriptome sequencing reads were assembled according to the S187 genome annotation file. NCBI OrfFinder was used to translate the assembled sequences into six frames. The constructed S187 SEP database was used for spectral matching and DB searching. Data analysis and visualization of DDA and DIA data were performed using the Spectronaut 15 platform (Biognosys, Wagistrasse, Switzerland), PEAKS studio (Bioinformatics Solutions Inc., Waterloo, Canada) and the R statistical framework. DDA MS raw files were analyzed using Spectronaut 15 and PEAKS studio, and peak lists were searched against the protein database. The data extraction and extraction window were set to “dynamic” with correction factor 1. Identification was established with a “normal distribution *P*-value estimator” and a q-value cut-off of 0.01. The profiling strategy was set as “iRT profiling,” with a q-value cut-off of 0.01.

### Function prediction analysis for SEPs

Clusters of Orthologous Groups (COGs), Gene Ontology (GO), and Kyoto Encyclopedia of Genes and Genomes (KEGG) databases were used for protein classification, homologous protein function, and metabolic pathway analyses, respectively. Based on related species, probable interacting partners were predicted using the STRING-db server to predict protein-protein interactions. The enrichment pipeline was used to perform GO and KEGG enrichment analyses. PSIPRED was used for secondary structure prediction with the data type sequence; the analytical methods of choice were PSIPRED 4.0 and MEMSAT-SVM (52, 53). An online version of the hmmscan program was used to identify SEPs with functional domains. InterProScan was used to search for the functions of non-annotated proteins (54). SignalP (55) was used to predict signal peptides of SEPs using parameters for Gram-positive bacteria. TMHMM (56) was run with default parameters to predict transmembrane sequences in SEPs.

### *De novo* assembly algorithms for identification of non-observed SEPs

PEAKS Studio version 10.6 was used to reanalyze mass spectral data. PEAKS was used with the following parameters: no digesting enzyme, fragment ion mass tolerance of 0.02 Da, parent ion tolerance of 7 ppm, and oxidation (M), acetylation (protein N-term), and deamidation (NQ) as variable modifications. Peptides were filtered using −10logP ≥ 20. Peptides with an average local confidence (ALC) ≥80% and without post-modification were filtered to obtain highly credible novel peptides. The results were mapped to the full protein library, which was created based on a six-frame translation library of the genome and transcriptome OrfFinder database of S187 using the peptide sequence matching software PeptideMapper (57). Mapped peptides were submitted to UniProt to search for and filter reported proteins. The parameters were set as follows: the searched species were limited to *Actinobacteria* (taxonomy: 201174), and leucine and isoleucine were considered equivalent.

Another novel peptide pipeline analysis was performed. The redundant parts of *de novo* peptides were deleted using BLASTp against the *Actinobacteria* sequence from the Nr database. The results were mapped to the S187 genome using tBLASTn, and the PAM30 scoring matrix and best matches were set. Peptides with identity and coverage ≥80% and an E-value ≤ 1 were detected. Following this, an ID lookup comparison, including calculated peptide location and ORF position, was carried out according to the annotated ORFs in chromosomes. An ORF length of less than 100 aa was used. Reported homologous sequences were filtered using the online tool BLASTp and the NCBI NR database. Peptide sequences with ALC values ≥80%, length ≥7, and mismatch numbers less than 2 aa were selected. Higher-quality spectra were filtered by matching less impure peaks and three pairs of b/y ions.

### New transcripts mining and ncRNA analysis

Mapped reads from the RNA-seq data set were assembled into transcripts in a reference annotation-based transcript assembly mode in two ways by using StringTie (v.2.2.1; Center for Computational Biology, Baltimore, USA) and Cufflink (v.2.2.1; Seattle, USA). Putative transcripts were obtained with the parameter “-m 30” in StringTie, while the parameter “--min-frags-per-transfrag 1” was set in Cufflink. Cuffcompare (v.2.2.1; Seattle, USA) was used to identify the location relationship between new transcripts and annotated genes. Then, transcripts with a length of >500 nt were excluded. All new transcripts were BLASTx with SEPome I and SEPome II. db_gencode was selected as codon 11, E-value was selected as 1e-5, and both similarity and coverage >80% were considered matches. All the candidate new RNAs were translated into amino acid sequences considering six frames, and the *de novo* only peptide was compared with ncRNA by BLASTp. At the same time, the task was selected as blastp-short, the scoring matrix was chosen as PAM30, and the E-value was selected as 10.

### Construction of NagE mutants

pSET152 *ermE* ^*^ derivative reporter vectors were constructed using PCR to amplify the sfGFP fragment. A different set of primers was used to amplify two sfGFP fragments: the complete sequence and the sequence with the start codon deleted. The resulting PCR fragment was cloned into the same sites as pSET152 *ermE* ^*^, and psfGFPwt and psfGFPmut were constructed. psfGFPmut-NagE-S187 and psfGFPmut-NagE-M145 were constructed by amplifying the NagE fragment without stop codons, and the PCR fragments were cloned into the same sites as psfGFPmut. All the resulting fusion genes were confirmed using sequencing. To construct overexpression strains, a PCR-generated DNA fragment containing the SEP-NagE coding sequence was cloned into the plasmid pSET152 ermE^*^. The primers used in this study are listed in Table S2. Restriction enzymes, PrimeSTAR Max DNA Polymerase, and In-Fusion HD Cloning Kits were purchased from TaKaRa Bio (Dalian, China). Fluorescent expression and overexpression plasmids were introduced into *E. coli* ET12567/pUZ8002 for conjugation with S187 and *S. coelicolor* M145. Exconjugants were plated on A1 solid medium (containing 50 µg/mL apramycin) and grown at 28°C. PCR amplification and DNA sequencing were used to determine the mutant genotypes.

### Observation of strain phenotype

Aerial mycelia were scraped and spread on a glass slide for observation under a fluorescence microscope (Zeiss, Jena, Germany) to observe the fluorescence phenotype. Fluorescence images were transferred to a computer and edited with ZEN 3.5 (blue editor) to achieve uniform brightness and contrast in group photos. To observe growth conditions, 3 × 10^7^ spores of each strain were plated on MS and TSB solid media in triplicate. The plates were incubated at 28°C for 3 and 5 days and observed every 12 hours. Observations were made using a Nava NanoSEM 450 scanning electron microscope (FEI Company, USA).

### Association analysis for bioactive metabolites

Fermentation broth (100 mL) was extracted using ethyl acetate (100 mL) and evaporated to dryness. The extracted products were analyzed using an UltiMate 3000 HPLC with UV detection at 280 nm. The HPLC system used methanol as eluent A and water as eluent B (water). The solvent gradient was set as follows: 15%–50% B, 12 minutes; 50%–80% B, 12 minutes; 80%–90% B, 10 minutes; 90%–100% B, 10 minutes. The samples were injected into a Luna Omega 3 µm Polar C18 (Phenomenex, USA; 100 × 2.1 mm) column and analyzed using TripleTOF 6600+ (AB SCIEX, USA) under the following LC analysis conditions: PDA detection wavelengths of 210, 254, and 280 nm. The acquired mass spectrometry data were uploaded and compared with global natural product social molecular networking (GNPS) (58). The data generated from GNPS for secondary metabolites was visualized using Cytoscape 3.9.1. The classic pathway was selected for anti-complement activity analysis based on previous work (59). The anti-complement activity was determined as the mean of triplicate measurements at each concentration.

### RNA isolation and real-time-quantitative PCR (RT-qPCR)

Total RNA was isolated using HiPure Bacteria L RNA (Magen, Guangzhou, China) and on-column digestion to remove DNA contamination. Complementary DNA (500 ng) was synthesized using M-MLV reverse transcriptase (TaKaRa Bio., Dalian, China). The *hrd* B gene was used as a reference to normalize the relative expression of S187. RT-qPCR was performed on a Bio-Rad system using 2× SYBR qPCR Master Mix (Vazyme Biotech Co., Nanjing, China). All data represent the mean ± SD of at least three biological replicates per condition; *P* < 0.05 was considered significant.

## RESULTS

### Sensitive streamlined platform

**Fig 1:**
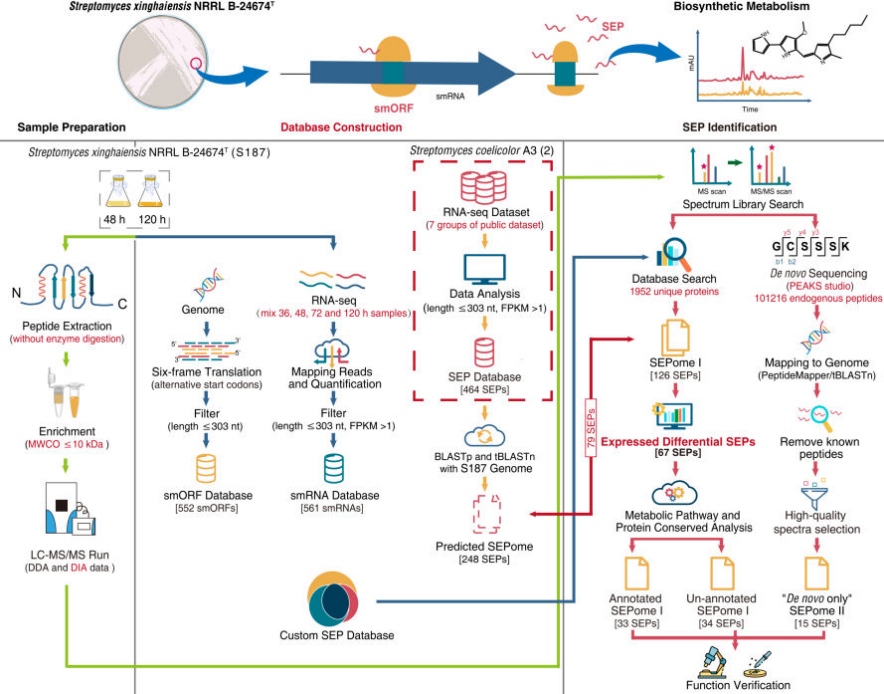
Integrated research strategy for discovery and identification of SEPs. Proteins were enriched without digestion and directly analyzed using liquid chromatography tandem mass spectrometry to obtain high peptide coverage. We searched the raw data using a customized database, including smORFs from S187 six-frame translation, a model strain smRNAs database, and S187 RNA-seq data to identify SEPs. *De novo* sequencing mapping of the genome was carried out to define ORFs and discover more novel SEPs. The steps in red font represent the optimized strategy for a comprehensive search of SEPs.

**Fig 2:**
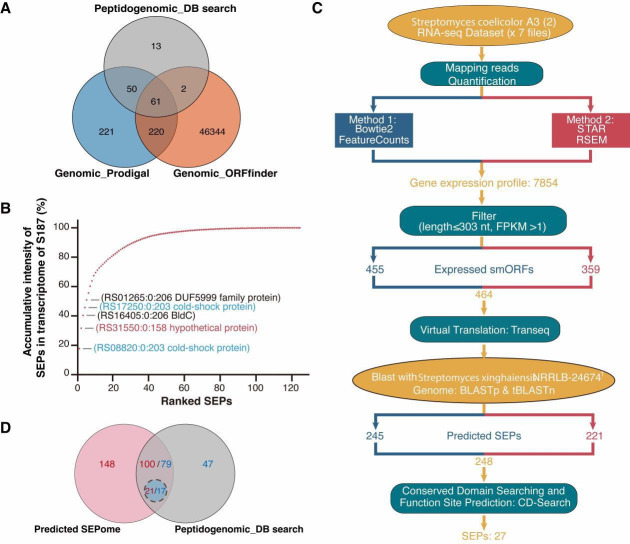
Construction of a predicted smORF database. (**A**) Venn diagram comparing results from OrfFinder and Prodigal based on the six-frame translation of the S187 genome and 126 SEPs from the peptidogenomic database. (**B**) Cumulative intensity of 126 SEPs in the S187 transcriptome. The top five most abundant smRNAs are labeled in the panel. Among these, smRNA coding cold shock proteins shared with the *S. coelicolor* A3 (2) predicted database are marked in blue, and the smRNAs coding a full-length coverage peptide is marked in red. (**C**) Processing of seven RNA-seq data sets from public databases of *S. coelicolor* A3 (2). (**D**) Overlap data of SEPs identified from the predicted SEPome and SEPome I databases. Blue circles indicate shared SEPs with functional sites.

An optimized and streamlined platform was proposed to improve the sensitivity of SEP identification (Fig. 1). Instead of the prevailing bottom-up strategy of proteomics (60, 61), we used direct extraction and enrichment of endogenous peptides with a 10 kDa molecular weight cut-off to increase the number of high-sequence-coverage peptides and identify more native peptides. The average coverage of SEPs was 39.8%, and four full-length coverage peptides were detected. Premium-quality custom databases are critical for the successful identification of SEPs. To address the challenge of inadequate publicly available databases, we created customized databases, including a genomic six-frame translation smORF database (Fig. 2A), a SEP database based on RNA-seq for S187(Fig. 2B), and an additional SEP database from seven sets of public high-quality transcriptome data of *S. coelicolor* A3 (2) (Fig. 2C, Fig. S1AB). All 27 sequences were functional SEPs with predicted conserved structural domains and functional sites (Table S3). We reached 86.5% coverage of SEPs from peptidogenomics, combined with the other two omic approaches. This multi-integrated approach was thus efficient and accurate for the discovery and identification of SEPs in strain S187. We used *de novo* sequencing, a database-independent approach to compensate for the limitations of DB searches, to mine more novel peptides and identify 68 novel SEPs not observed in available Actinomyces databases or global public databases.

### Peptidogenomic landscape based on DB search

**Fig 3:**
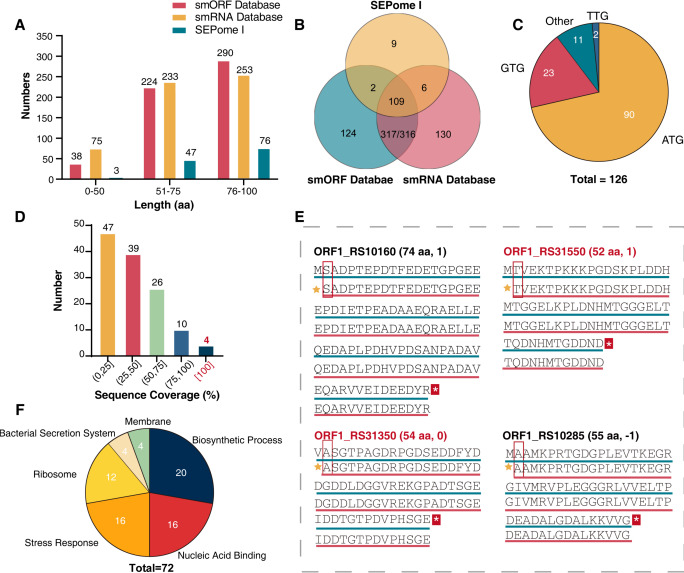
Peptidogenomic database search results. (**A**) Sequence length analysis of SEPs in three databases. (**B**) Number analysis of SEPs in three databases (genomic database: green circle; RNA-seq database: red circle; peptidogenomic database: yellow circle). (**C**) Utilization rate of initial translation with canonical (ATG, CTG, GTG, and TTG) and other start codons of the peptidogenomic database. (**D**) Sequence coverage analysis of peptidogenomic database. (**E**) Coverage comparison of four full-length peptides (red line) with protein sequences (green line) in S187. Red rectangles indicate N-terminal methionine cleavage amino acids. Two unique SEPs in S187 are marked by red characters; 1/–1 is the differential expression of two time-points (48 and 120 hours). (**F**) Functional cluster of the 72 annotated SEPs using KEGG, GO, and InterProScan analysis.

Based on global *Streptomyces* and custom database profiling, 24,155 peptides were identified, including 1,952 unique proteins. Among the 1,952 unique proteins, there were 126 polypeptides less than 100 aa, of which 76–100-aa SEPs comprised the majority (~60%), and 3 SEPs were under 50 aa (Fig. 3A). All 126 identified SEPs could be mapped to complete ORFs in the genome, which are recognized under the strict definition of SEP (62). The comparisons of the smORF database and smRNA database with SEPome I revealed that parallel analysis results from the three omics yielded 109 shared SEPs, with an 86.50% coverage of SEPome I (Fig. 3B). This suggests that rigorous criteria for evaluating the customized database significantly effect overall SEP identification.

Given the high G+C content of the *Streptomyces* genome, the canonical start codons ATG, GTG, and TTG are usually used (63). Here, 91% of the 126 smORFs started with canonical codons, although 11 started with other codons (Fig. 3C; Fig. S1C). High-coverage SEPs can provide high confidence in SEP identification and functional verification (64). The sequence coverage for almost one-third of the 126 SEPs was >50% (Fig. 3D). Moreover, four SEPs with full sequence coverage were detected, with only the N-terminal methionine or valine missing after mapping with the genome (Fig. 3E). N-terminal methionine cleavage is a general modification in the bacterial peptide biosynthetic process, usually leaving the second amino acid of the peptide, such as arginine, lysine, and leucine (65, 66). These results indicate that the non-digestion strategy significantly improves sequence coverage and enhances confidence in SEP identification.

SEPome I is involved in the dynamic transition from the exponential growth phase to the bioactive secondary metabolite biosynthesis phase. Therefore, we investigated the 126 identified SEPs for their role in metabolism using protein function prediction and metabolic pathway analysis. All 72 annotated SEPs could be divided into six groups: biosynthetic processes, nucleic acid binding, stress responses, ribosomes, bacterial secretion systems, and membranes (Fig. 3F; Table S4). Significantly, there were another 54 non-annotated SEPs in SEPome I, hypothetical proteins with no characterized function that had experimental validation (Fig. S1D). As SEPs are often associated with small membrane proteins and may have signal peptides (25), four novel and nine annotated SEPs were predicted to be secreted proteins with signal peptide sequences, whereas three single SEPs were predicted to be membrane proteins with a transmembrane structure (Table S5; Fig. S1E).

### Novel SEP identification using *de novo* sequencing

**Fig 4:**
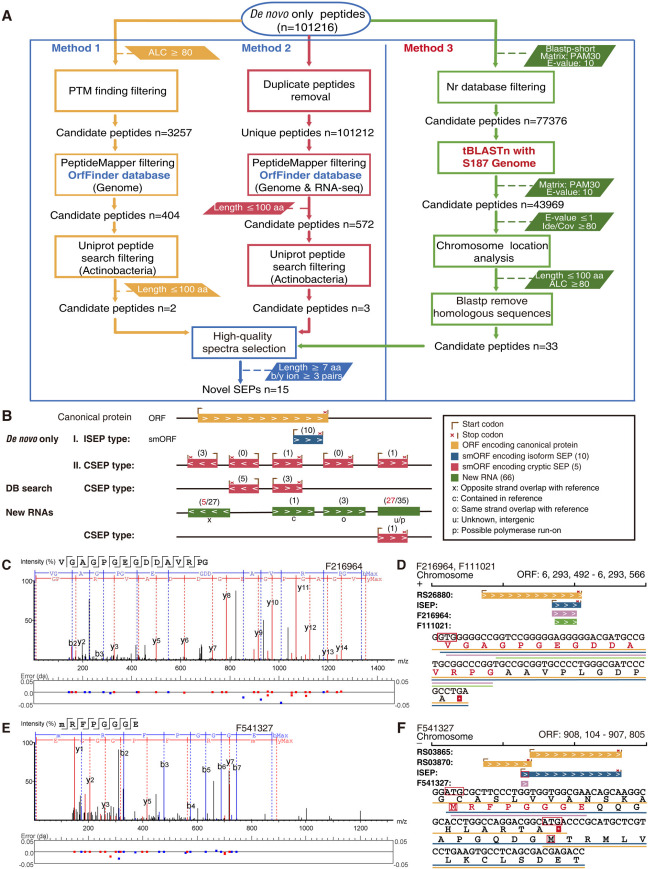
*De novo* sequencing identification of novel SEPs. (**A**) Illustration of three processes for analyzing “*de novo* only” data to identify novel SEPs. (**B**) Classification and characteristics of detected novel SEPs and new RNAs. Isoform SEPs are novel isoforms derived from a larger protein. Cryptic SEP is a SEP located in non-coding or non-annotated regions of the genome. Black numbers in brackets show the number of each type of SEP and new RNA. Red numbers in brackets show the numbers of ncRNAs. (**C**) MS_2_ spectrum of the novel peptide F216964, VGAGPGEGDDAVRPG. (**D**) Illustration of F216964 and F111021 locations on the S187 chromosome. The amino acid sequences of the novel peptides F216964 and F111021 are marked with purple and green lines, respectively. (**E**) MS_2_ spectrum of the novel peptide F541327, MRFPGGGE. (**F**) Illustration of F541327 location on the S187 chromosome. The amino acid sequence of the novel peptide F541327 is marked by a purple line.

Database-independent *de novo* sequencing is considered an alternate analytical method for SEP identification, whereby “*de novo* only” sequences comprise novel peptides, unknown modified peptides, or other molecules of interest (67). To mine novel SEPs outside the database, DDA and DIA data were reanalyzed using PEAKS studio as a complementary approach. Subsequently, three different methods were applied to identify novel SEPs (Fig. 4A). Five novel peptides with a length of less than 300 bases were acquired after removing candidate peptides corresponding to ORFs (Table S6). Then, 33 novel candidate peptides were selected according to Method 3. All 38 novel candidate peptides could be back-correlated to the corresponding small ORFs in the genomes, demonstrating the reliability of the analysis.

Additionally, 15 *de novo* only novel SEPs emerged after high-quality spectral selection (Table S6). The SEP locations appear to fall into two categories based on their positions relative to conventionally annotated proteins in the chromosome (Fig. 4B). Cryptic SEPs (CSEPs), like cryptic proteins, are distributed in ostensibly non-coding or non-annotated regions in the genome. Isoform SEPs (ISEPs) are located within the sequences of functional proteins, and a hidden start codon is used in translation, leading to the generation of a novel peptide isoform. However, the functional activity of the peptide differs from that of the co-located protein (68, 69). The five CSEPs are located on different ORFs from the annotated proteins in the genome annotation data; therefore, these are novel proteins that are not associated with existing annotated ORFs, providing complementary information for genome annotation. The ISEP peptide F131566 was detected in the DB search as the full-coverage sequence SEP RS31550, demonstrating the high accuracy of this approach for identification. The database-driven DB search approach can identify novel SEPs despite the limitations of the static search space.

More than three MS_2_ spectra within the peak matched the spectral database for peptide F216964 (VGAGPGEGDDAVRPG) (Fig. 4C). A possible start codon (GTG) was identified in the gene sequence for F216964, and gene structure analysis showed that this novel peptide was located on the positive strand and overlapped with the extended DNA sequence of the annotated gene RS26880 (6, 291, 884–6, 293, 566 bp) encoding FAD-dependent oxidoreductase. Nevertheless, the canonical protein translated by RS26880 was not detected in the protein database using the DB search strategy. Based on the stop-codon-to-stop-codon principle and the observed peptide data, this novel peptide, F216964, may map to a 24-aa ISEP translated from a hidden start codon in ORF RS26880 (Fig. 4D). The 24-aa ISEP was located at the C-terminus of the longer canonical protein and shared the same stop codon as RS26880. Another *de novo* only peptide, F111021 (GPGEGDDAVRPG), matched the same translation area as F216964 (Fig. S1F), confirming that this novel gene sequence encodes a 24-aa ISEP. Three spectra were detected for the peptide F541327 (MRFPGGGE) (Fig. 4E), which was located on the negative strand of the chromosome and overlapped with RS03870. F541327 and RS03870 were not encoded by the same ORF, although peptide F541327 was assigned to the C-terminus of RS03870 in the DB search. Evidently, F541327 does not belong to RS03870. A methionine residue encoded by one theoretical start codon (ATG) on F541327, and this novel peptide was mapped with a smORF that encoded a 99-aa ISEP (908, 104–907, 805 bp) (Fig. 4F). These results demonstrate that this ISEP containing a new translation start site is a novel peptide, providing complementary data for genome annotation. *De novo* sequencing, complementing to DB searches, provides a new approach to peptidomics.

### Potential SEPome of the model strain *S. coelicolor* A3 (2) and key bioprocess SEPs

**Fig 5:**
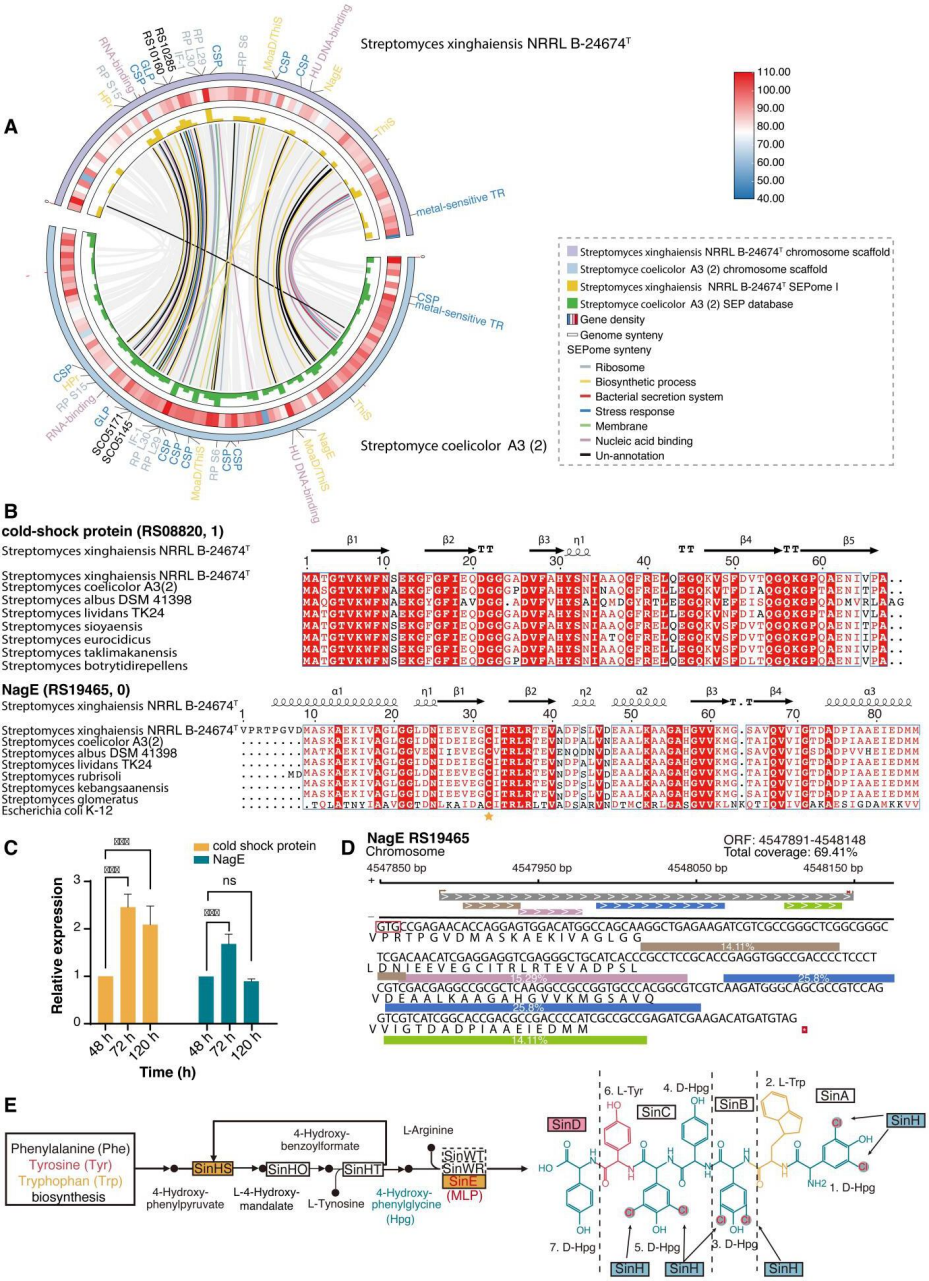
Conservation of SEPs in the *Streptomyces* strains. (**A**) Circos plot showing genome, transcriptome, and protein information for S187 and *S. coelicolor* A3 (2). Information on genome synteny, RNA-seq data, and genome annotation shown from the inside to the outside rings, with the outermost ring representing chromosomes. SEPs shared by S187 and *S. coelicolor* A3 (2) divided into six categories and marked by lines of different colors. SEPs containing functional sites are marked on the outermost ring and colored according to their functional description. (**B**) BLAST sequence alignment analysis and predicted secondary structures of NagE and cold shock proteins in other modal *Streptomyces* species and microorganisms. The conserved active sites are marked by a star. (**C**) RT-qPCR quantification results of cold shock protein (RS08820) and NagE (RS19465) at fermentation time points of 48, 72, and 120 hours relative to 48 hours for each gene analyzed. (**D**) Sequence coverage analysis of NagE with the four detected peptides. (**E**) Of the 1,952 global proteins detected in peptidogenomics, those involved in the xinghaimycin biosynthesis metabolic pathway were analyzed, including one SEP-MLP. Proteins with upregulated expression are marked in red, those with downregulated expression in blue, and proteins with unchanged expression in yellow.

Since *S. coelicolor* A3 (2) has become a model strain for genetics, development, and antibiotic production in the genus *Streptomyces*, we explored the potential SEPs in this workhorse strain. A set of 464 smRNAs was obtained from public transcriptome data sets of *S. coelicolor* A3 (2), of which 248 smRNAs coding SEPs were observed in the predicted SEPome of S187. Additionally, 79 SEPs were observed in SEPome I of the S187 peptidogenomic, with 62.7% crossover between the two databases (Fig. 2D). Additionally, there are 59 annotated and 20 non-annotated common SEPs (Fig. 5A). Ka/Ks values (70) were all below 1, demonstrating that these SEPs are under purifying selection pressure, leading to conservative evolution in these two species. A further 21 SEPs with conserved structural domains and functional sites in *S. coelicolor* A3 (2) were mapped to 17 functional SEPs of strain S187 (Table S3). These common SEPs may play significant roles in the biological processes of *Streptomyces*.

SEPs usually contain more α-helices than other protein secondary structures (71). As expected, most of the 17 functional SEPs had predicted α-helical structures, suggesting that these SEPs may play roles in signal transduction by interacting with the cell membrane (Fig. S2A). Cold shock proteins have been reported to be smORFs, and share a five-stranded β-barrel structure (72, 73). This simple protein structure allows cold-shock proteins to associate with DNA and RNA strands, resulting in various biological functions, such as regulating global gene expression and influencing signal transduction pathways under stress conditions (74). smRNA coding cold-shock protein represent the top two cumulative intensities in transcripts of *S. coelicolor* A3 (2) and match the top one (RS08820) in the S187 transcriptome (Fig. S2B). The cold-shock protein RS08820 in the S187 strain showed a high sequence similarity with functionally characterized SEPs in other *Streptomyces* species (Fig. 5B). The RT-qPCR results showed expression changes during periods of secondary metabolism (Fig. 5C; Fig. S2B), showing that this SEP carries out essential physiological functions.

PTS^Nag^, a PTS system using NagE as a conserved sucrose/glucose PTS IIB domain, is a biased carbon source uptake pathway for transporting N-acetylglucosamine (GlcNAc) into streptomycete cells (75, 76). The PTS system proteins PstH and NagE were common SEPs of *S. coelicolor* A3 (2) and S187. Four unique peptides detected in the mass spectrometry data were matched with different sequences of NagE, with a coverage of up to 69.41%, reflecting the accuracy of identification for this 86-aa SEP (Fig. 5D). Furthermore, NagE exhibited high conservation with functionally characterized SEPs in other *Streptomyces* species and many industrial strains producing active metabolites (Fig. 5B). The expression of Nag*E* changes during the period of secondary metabolism (Fig. 5C; Fig. S2C).

MbtH-like protein (MLP), which affects the production of non-ribosomal peptide (NRP) compounds by initiating and enhancing enzymatic adenylation activity (77 – 80), was detected in the SEP database of *S. coelicolor* A3 (2) and SEPome I of the S187 strain. Moreover, MLP exists in many pathogenic bacteria, such as *Mycobacterium tuberculosis* and *Pseudomonas aeruginosa*, and industrial strains with active metabolites, such as the vancomycin-producing strain A*mycolatopsis orientalis*. These MLPs exhibit high sequence conservation with three tryptophan active enzyme sites (Fig. S2D). Moreover, MLP is encoded by the gene *sin*E in the BGC for xinghaimycin and plays a vital regulatory role in its biosynthesis in S187 (Fig. 5E; Fig. S2E). We observed that MLP may directly interact with the NRP synthetases (*sin*A-*sin*D) in the xinghaimyxin BGC and regulate xinghaimycin biosynthesis in S187 (Fig. S2F).

### Peptidogenomic association analysis of metabolic processes

**Fig 6:**
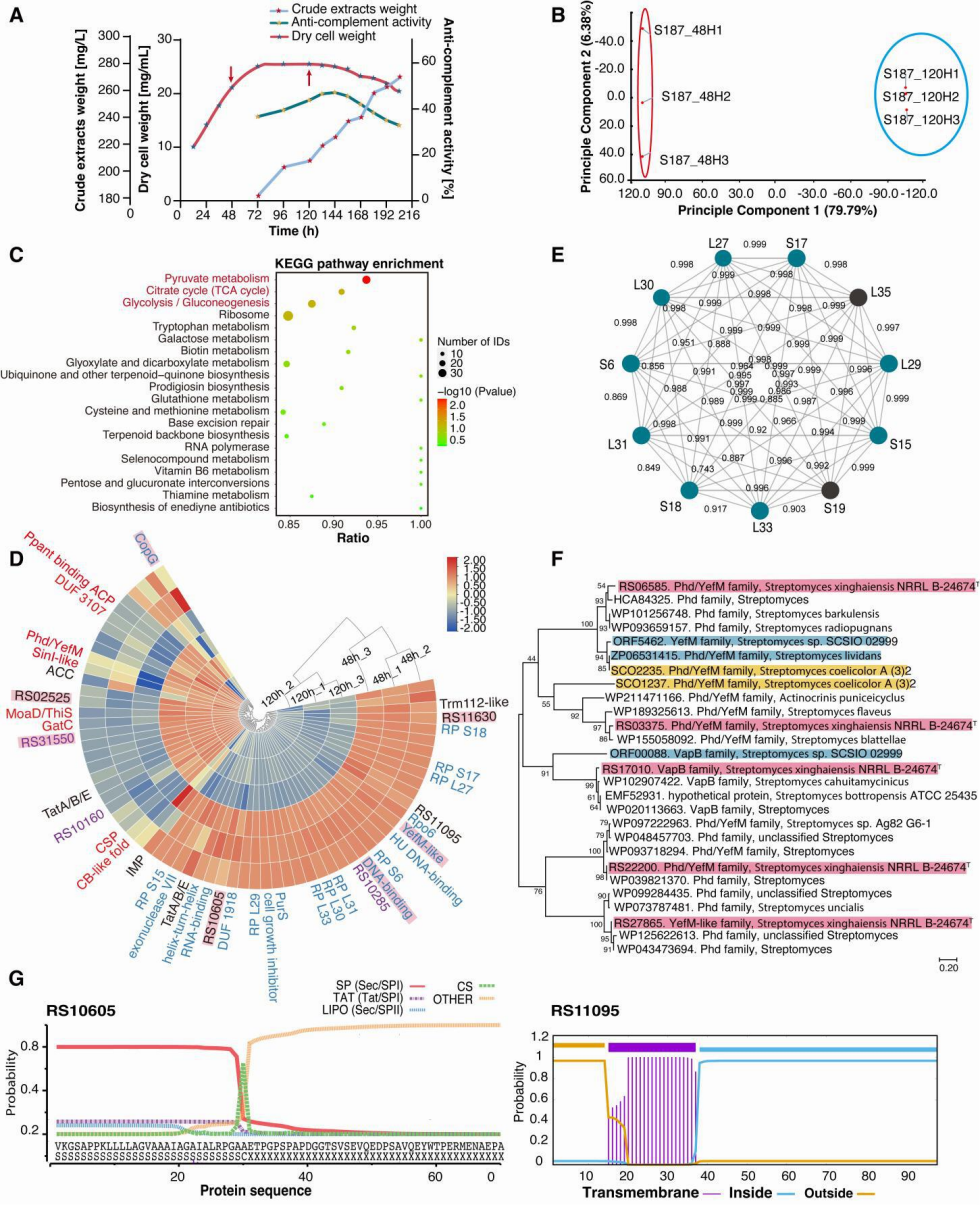
Analysis of SEPs according to metabolic processes. (**A**) Growth and anti-complement activity curve of S187. Each point represents the mean ± standard deviation of three independent cultures. Arrows indicate time points for sample collection for peptidogenomic analyses. (**B**) Principal component analysis of all quantifiable proteins at the two time points. The results show excellent proteomic separation between the two groups. (**C**) KEGG pathway enrichment analysis of 798 significantly differentially expressed global proteins (*P* > 0.05). Differentially expressed proteins are mainly enriched in carbohydrate-metabolism-related pathways. (**D**) HeatMap of differentially expressed SEPs between 48 and 120 hours during S187 fermentation. Three replicates for each group were normalized for different proteins and clustered. Up-regulated proteins are marked in red, down-regulated in blue, membrane-associated proteins in black, and full sequence coverage proteins in purple. S187 unique proteins are marked by pink rectangles. (**E**) Analysis of ribosomal protein interaction mapping. Altered proteins were included in the protein interaction network using the STRING database (combined score >0.85). (**F**) Evolutionary tree of *Streptomyces* antitoxin proteins. The five identified antitoxin proteins of S187 are marked by pink rectangles, the shared antitoxin proteins of *S. coelicolor* A3 (2) by a yellow rectangle, and antitoxin proteins characterized in *Streptomyces* by blue rectangles. (**G**) Identification of signal peptide functionality and cleavage sites within signal peptides. Diagram showing that RS10605 may be a secreted protein. One transmembrane helices region (purple) is in RS11095, indicating association with a membrane protein.

The secondary metabolites of the S187 strain are diverse, and anti-complement activity was observed. Sample collection time points of 48 and 120 hours were chosen to assess expression levels of endogenous peptides and SEPs during growth and secondary metabolism (Fig. 6A and B). Results for significant enrichment of the 1,952 unique proteins from peptidogenomics showed that carbohydrate and energy production pathways play important roles in S187 metabolism (Fig. 6C). Moreover, 798 unique proteins exhibited significant differential expression during the secondary metabolism phase of the S187 strain (Fig. S2G).

We explored 67 significantly altered SEPs to investigate associations between SEPs and metabolites of *Streptomyces* (Fig. 6D). Ribosome-associated SEPs were the most highly enriched and represented the most functional groups (Fig. S2H). All 11 ribosome SEPs including 9 downregulated SEPs, interacted with each other and showed close relationships (Fig. 6E), indicating that these ribosome-associated SEPs may be mutually connected and jointly influence secondary metabolite biosynthesis (81, 82). The type II toxin-antitoxin (TA) system plays a key role in various *Streptomyces* species regarding physiology, environmental stress responses, and antibiotic synthesis (83 – 85). Two significantly altered YefM family SEPs belonging to the TA system were identified. Phylogenetic evolutionary relationship analyses revealed three unique antitoxin SEPs (RS22200, RS17010, and RS27865) belonging to the S187 strain that showed different clades in phylogenetic evolutionary relationships as novel TA systems (Fig. 6F). Therefore, many antitoxin SEPs were detected during the secondary metabolite phase of strain S187 and may be associated with its growth in extreme deep-sea environments.

Small peptides usually contain a single structural domain and can interact with larger proteins to fine-tune complicated biological systems (86). Canonical signal peptide sequences and transmembrane structures were observed in annotated functional SEPs and in non-annotated SEPs (Fig. 6G). These abundant unknown SEPs, not been identified in previous databases, are likely to have important biological functions.

### Identification of a potential growth-affected factor, NagE, associated with metabolism in *Streptomyces*

**Fig 7:**
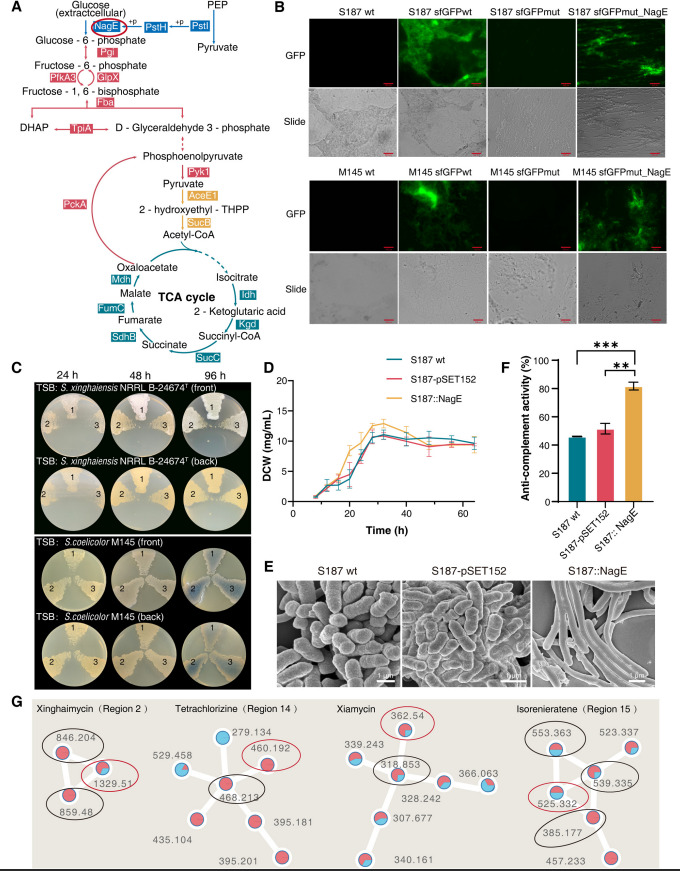
Expression verification and functional validation of the SEP of NagE. (**A**) Of the 1,952 global proteins detected in peptidogenomics, those involved in carbohydrate-metabolism-related pathways including the PTS system (blue), pyruvate metabolism (yellow), TCA cycle (green), and gluconeogenesis (red), were analyzed. The S187 core carbon metabolism pathway and major enzymes are shown. Pgi, glucose-6-phosphate isomerase; PfkA3, 6-phosphofructokinase; GlpX, fructose 1,6-bisphosphatase II; Fba, fructose-bisphosphate aldolase; TpiA, triosephosphate isomerase; PckA, phosphoenolpyruvate carboxykinase; Pyk2, pyruvate kinase; AceE1, pyruvate dehydrogenase subunit E1; SucB, 2-oxoglutarate dehydrogenase; Idh, isocitrate dehydrogenase; Kgd, alpha-ketoglutarate decarboxylase; SucC, succinyl-CoA synthetase subunit beta; SdhB, succinate dehydrogenase iron-sulfur subunit; FumC, fumarate hydratase; Mdh, malate dehydrogenase. (**B**) sfGFP fluorescence was used to confirm the expression of SEP NagE in S187 and M145 cells. The scale bar represents 500 µm. (**C**) The colony of wild type and *nag*E over-expression mutants of S187 and M145 on TSB solid medium after 24, 48, and 96 hours. 1: S187/M145::NagE; 2: S187/M145-pSET152; 3: S187/M145 wt. (**D**) Growth curves of wild type and *nag*E over-expression mutants of S187 cultures cultivated in TSB liquid medium. Each point represents the mean ± standard deviation of three independent cultures. (**E**) Scanning electron micrographs of the S187 wt, S187-pSET152, and S187::NagE mutants. Scale bars represent 1 µm. The strains were grown on TSB liquid medium and imaged after 20 hours. (**F**) Anti-complement activity of S187::NagE, S187-pSET152, and S187 wt crude extracts (^**^ *P* <0.01; ^***^ *P* <0.001). (**G**) GNPS analysis for the S187::NagE (red dot) and S187-pSET152 (blue dot) fermentation products. The compounds represented by the four clusters are misaugamycin, tetrachlorizine, Xiamycin, and isorenieratene.

According to peptidogenomic results, pyruvate metabolism, the TCA cycle, and glycolysis/gluconeogenesis were the top three enriched KEGG pathways of significantly differentially expressed proteins during active metabolism in the S187 strain. All these genes are related to glucose metabolism. We noted that one of the SEPs, NagE, is responsible for glucose transport at the beginning of gluconeogenesis, affecting the top three pathways (Fig. 7A, Fig. S3A). Then, we further confirmed the intracellular expression of NagE in *S. coelicolor* M145 and S187 (Fig. 7B, Fig. S3BC). The overexpression mutant S187::NagE formed spores earlier, and the color of the colony was darker than that of the wild-type strain (S187 wt) and comparison strain (S187-pSET152) on TSB plates (Fig. 7C, Fig. S3D). M145::NagE showed no significant difference in spore growth. However, the colony appeared to have a different color than that of the control groups on TSB plates. The same result was observed on plates based on different media (Fig. S3E).

Bioaccumulation of S187::NagE was higher than that of the control strain in the logarithmic phase, and the curve of the mutant strain showed an earlier inflection point at 24 hours (Fig. 7D). However, a similar result was not seen in the growth curve of *S. coelicolor* M145 (Fig. S3F). NagE affects strain growth and participates in morphological differentiation in S187, not in *S. coelicolor* M145 (Fig. 7E). However, the change in color of M145::NagE colonies on plates with different media showed that NagE influences the metabolism of two main antibiotics, prodiginine and actinorhodin, in *S. coelicolor* M145. We observed that the anti-complement activity of fermentation extracts from S187::NagE was significantly increased relative to that of wild-type S187 (Fig. 7F). The production of secondary metabolites increased, and a few new compounds (87) appeared in S187::NagE (Fig. 7G; Fig. S3G). In particular, the production of xinghaimycin (*m/z* 1,329.51) increased, and two potential intermediates (*m/z* 846.204 and *m/z* 859.48) appeared in the metabolic products of S187::NagE. Thus, NagE appears to be involved in the production of secondary metabolites and primary metabolism in strain S187.

## DISCUSSION

*Streptomyces* species have the potential to synthesize various bioactive metabolites for applications in agriculture, medicine, and food technology (88, 89). However, the underlying control mechanisms in *Streptomyces* metabolic pathways remain unclear. To identify novel SEPs and determine pathways, we employed a multi-integrated approach involving comprehensive database building, peptidogenomics, and *de novo* sequencing. We identified 140 SEPs in *Streptomyces*, including 68 novel SEPs (54 non-annotated from DB searches and 15 *de novo* only) and at least 67 SEPs predicted to be closely related to secondary metabolism. For the multi-integrated approach, 63 and 111 SEPs were predicted using OrfFinder and Prodigal (90), respectively. The predicted data simultaneously obtained using these two methods covered almost half of the 126 SEPs detected using peptidogenomics, revealing the accuracy of the combined data. However, prokaryotic SEPs of up to 100 aa can be included, considering the complex metabolism of *Streptomyces* (16, 21, 91). In this study, we set a cut-off of 100 aa for the size of candidate proteins and obtained comprehensive protein structural and annotation information for SEPs affecting S187 metabolism. SEPs of less than 50 aa comprised 2.38% of SEPome I and 46.67% of SEPome II.

Due to the lack of available non-model biological databases, the identification of SEPs in strain S187 was arduous. A database-independent *de novo* sequencing approach is used for discovering novel SEPs. The combined strategy of analyzing *de novo* sequencing data and strict selection criteria for candidate SEPs further improves the quantity and reliability of the identified SEPs (92 – 94). smORFs located in non-coding regions can have functions relating to stress responses, virulence, and transport, and novel isoforms of canonical proteins can have important functions (68, 95). However, such ORFs are often ignored in database-dependent analyses. None of the five CSEPs or four ISEPs were observed in the custom database, an outcome that provides support for the DB search approach. These novel SEPs have not been annotated in the S187 genome. Another six ISEPs were observed in the custom database and not detected as SEPs in the DB search because of the limitations of this database-driven approach. These results demonstrate the advantage of database-independent *de novo* sequencing in identifying SEPs in proteomics. Combining both approaches has the capability and possibility to discover new rare genes and novel SEPs.

Meanwhile, we explored the new transcripts and 66 new RNAs that are independent of the most updated gene annotations from genomics in the RNA-seq database. Based on the genome location, new RNAs were classified into the following types (96, 97): 5 “u” (intergenic, unknown), 30 “p” (RNA within 2000 nt of annotated genes and not overlapping), 27 “x” (opposite strand overlaps with reference), 3 “o” (on the same strand overlap with reference), and 1 “c” (contained in reference) (Fig. 4B). Moreover, 19 new RNAs that could be matched with peptide segments of MS/MS data were codable. ncRNAs are critical regulatory elements that control various cellular processes, including regulating primary metabolism, stress response, morphological differentiation, and secondary metabolism (98 – 101). Thirty-two ncRNAs were observed in the 66 new RNAs. Noteworthy, four ncRNA-encoded peptides, which one encodes for a 61 aa CSEP, were observed (Fig. 4B). This supports the conclusion that ncRNAs can be translated into peptides and even, rarely, SEP (102).

Many SEPs related to metabolism were identified in this study (Fig. S4), including 47 unique SEPs of S187 and 79 common SEPs with *S. coelicolor* A3 (2) in SEPome I based on the peptidogenomics of strain S187. We further surveyed the common SEPs in two other model strains, *S. lividans* TK24 and *S. albus* NRRL B-1811^T^, and observed 74 universal strains. In unique SEPs, 13 were functionally annotated (Table S7). In addition, 12 SEPs, including four novel ones located on 9 BGCs, were closely associated with regulating and producing the main bioactive secondary metabolites and transferring substances in S187 (Table S8). Xinghaimycin is a typical S187 metabolite exhibiting anti-complement activity, similar to vancomycin, a glycopeptide antibiotic (103). KEGG analysis of the peptidogenomics of S187 showed that MLP is involved in the synthesis pathway of vancomycin and xinghaimycin. Separate from MLP, three canonical proteins, SinHS, SinD, and SinH, related to 4-hydroxyphenylpyruvate dioxygenase, NRP synthetase, and tryptophan 7-halogenase, respectively, were detected in peptidogenomics. Another 56 potential smORFs were located at the margin between functional genes in the BGC of xinghaimycin, and 13 of these were identified in the smORF database searched using OrfFinder (Table S9).

Different carbon sources (such as glucose, fructose, mannose, and GlcNAc) are phosphorylated in the endoplasmic reticulum by bacteria through the PTS system. The PTS system regulates nitrogen metabolism, mediates the homeostasis of iron and potassium, and is involved in stress responses (104 – 106). In this study, NagE significantly affected the production of secondary metabolites in S187 and *S. coelicolor* M145. Additionally, it was involved in the growth, development, and sporulation of S187. However, a similar phenomenon was not observed during the growth of *S. coelicolor* M145. Prediction of protein structure showed that S187 NagE has an α-helical structure in the N-terminal region (Fig. S3H), which should lead to a more stable protein (107, 108). Oxidative metabolism is considered the primary mechanism in *S. coelicolor* because of antibiotic (actinorhodin) synthesis (109, 110). This might be the reason for the lack of a significantly different phenotype in the *nag*E overexpression strain of *S. coelicolor* M145. We observed that the production of two major secondary metabolites, xinghaimycin, and xiamycin, was increased, and the production of other potential intermediates was stimulated by the overexpression of *nag*E in S187. The approach used in this study increases the feasibility of active metabolite identification and potentially reveals biosynthetic processes.

In summary, the database establishment and reanalysis of a model organism database in this study offer a rapid, cost-effective, and reliable approach to performing high-throughput sequencing or proteomics studies in other non-model organisms. The SEPs identified in this study can be exploited as valuable target SEPs to provide a comprehensive view of the mechanisms underlying secondary metabolism in *Streptomyces*. This research establishes a solid foundation for future applications investigating prokaryotic growth, differentiation, and secondary metabolism.

## Data Availability

The mass spectrometry proteomics data have been uploaded to the ProteomeXchange Consortium with the data set identifier PXD038189. The RNA-seq data have been uploaded to the National Center for Biotechnology Information (NCBI) database with accession code PRJNA67543.

## SUPPLEMENTAL MATERIAL

Figure S1 msystems.00245-23-s0001.tif smORFs smRNA, and SEP analysis.

Figure S2 msystems.00245-23-s0002.tif Analysis of metabolism-related SEPs.

Figure S3 msystems.00245-23-s0003.tif Effect of SEP NagE on growth and metabolism.

Figure S4 msystems.00245-23-s0004.tif Mining multidimensional analysis of metabolic-related SEPs.

Supplemental Material msystems.00245-23-s0005.docx Legends for Figures S1 to S4; Tables S1 to S9.

ASM does not own the copyrights to Supplemental Material that may be linked to, or accessed through, an article. The authors have granted ASM a non-exclusive, world-wide license to publish the Supplemental Material files. Please contact the corresponding author directly for reuse.
