# Supplementary figures and images for: Multi-integrated approach for unraveling small open reading frames potentially associated with secondary metabolism in Streptomyces
Source: mSystems. 2023 Sep 15;8(5):e00245-23. doi: 10.1128/msystems.00245-23 (PMC10654065; doi:10.1128/msystems.00245-23)

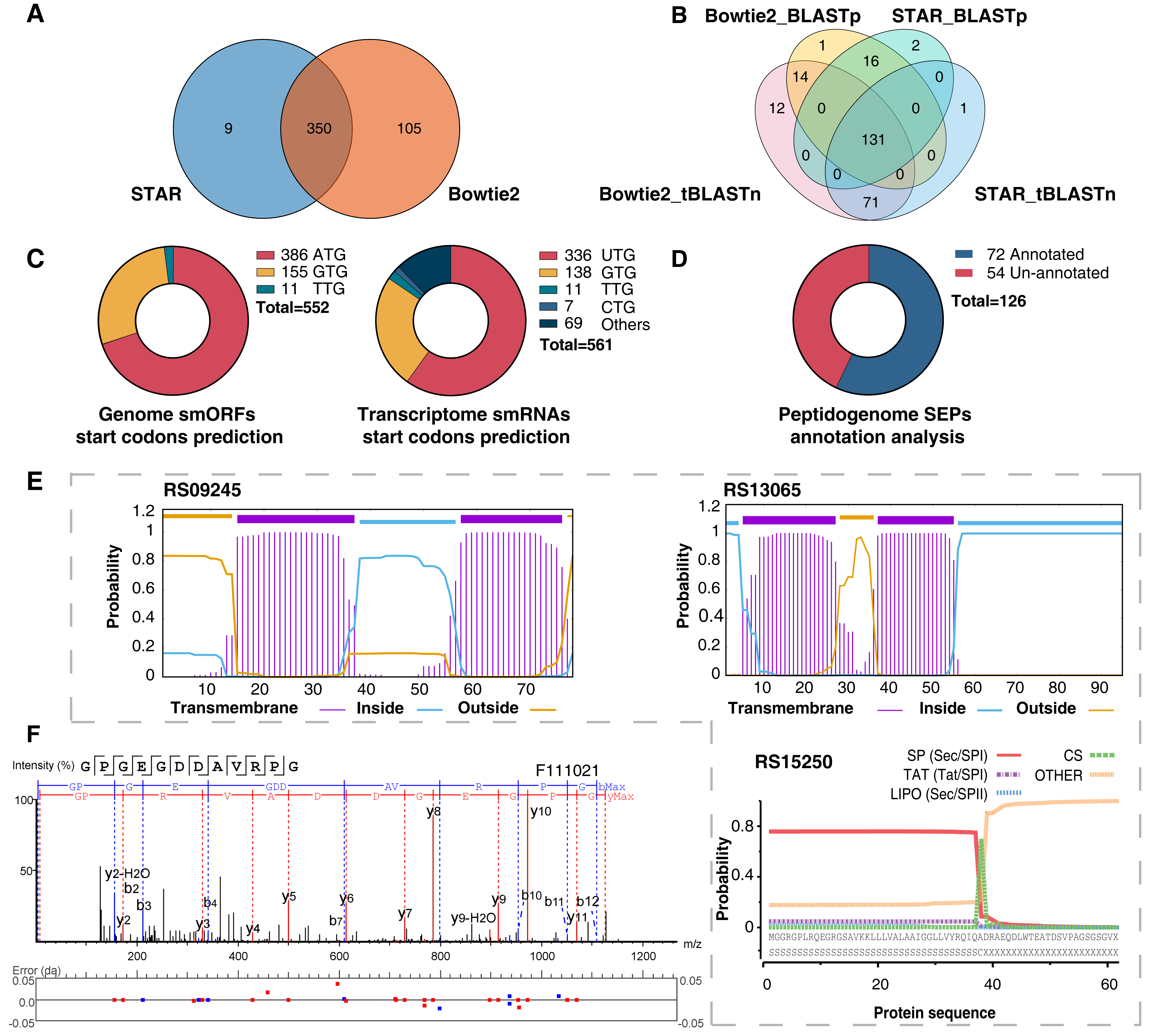

Supplement: Figure S1 — smORFs smRNA, and SEP analysis. [file msystems.00245-23-s0001.tif]

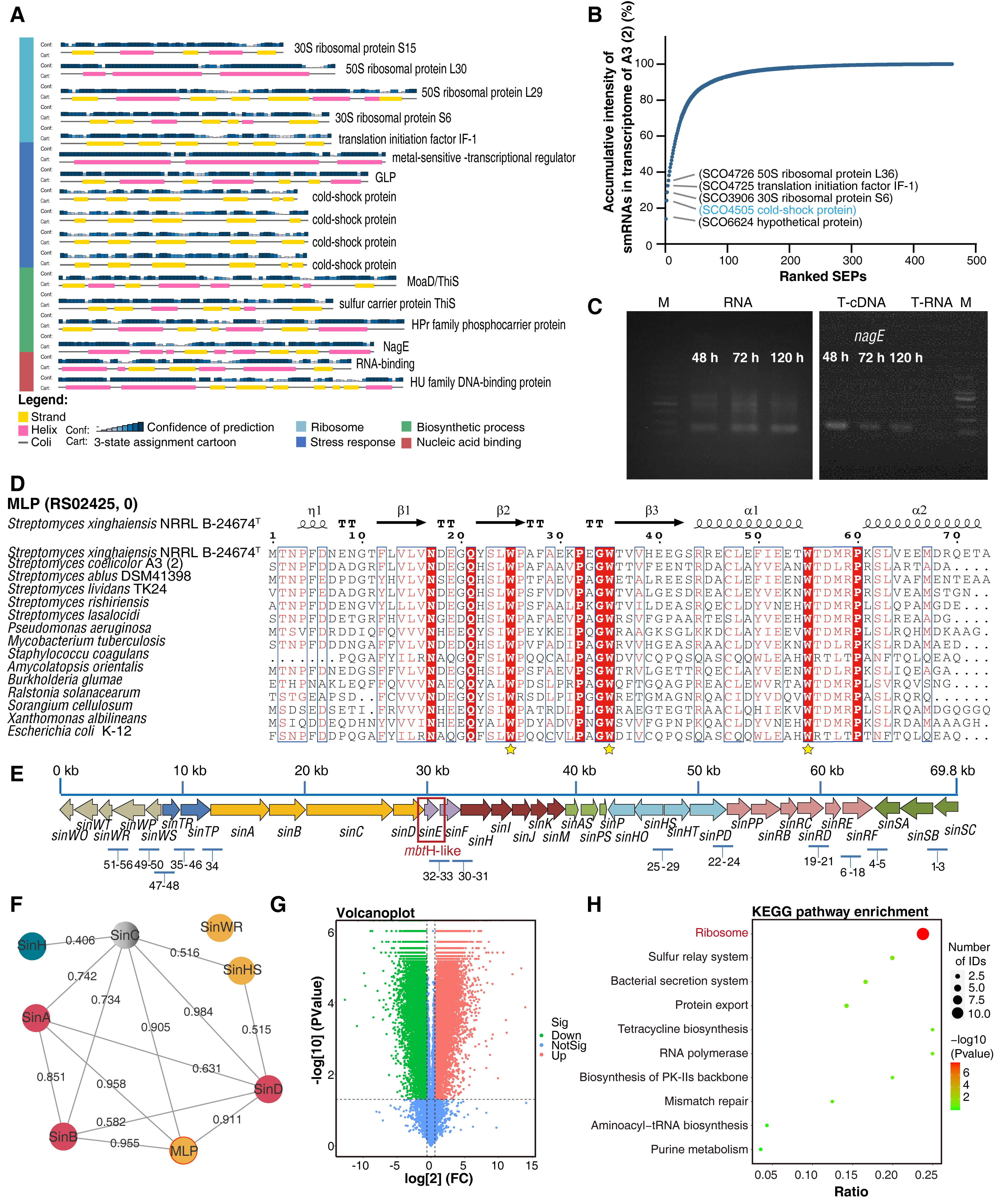

Supplement: Figure S2 — Analysis of metabolism-related SEPs. [file msystems.00245-23-s0002.tif]

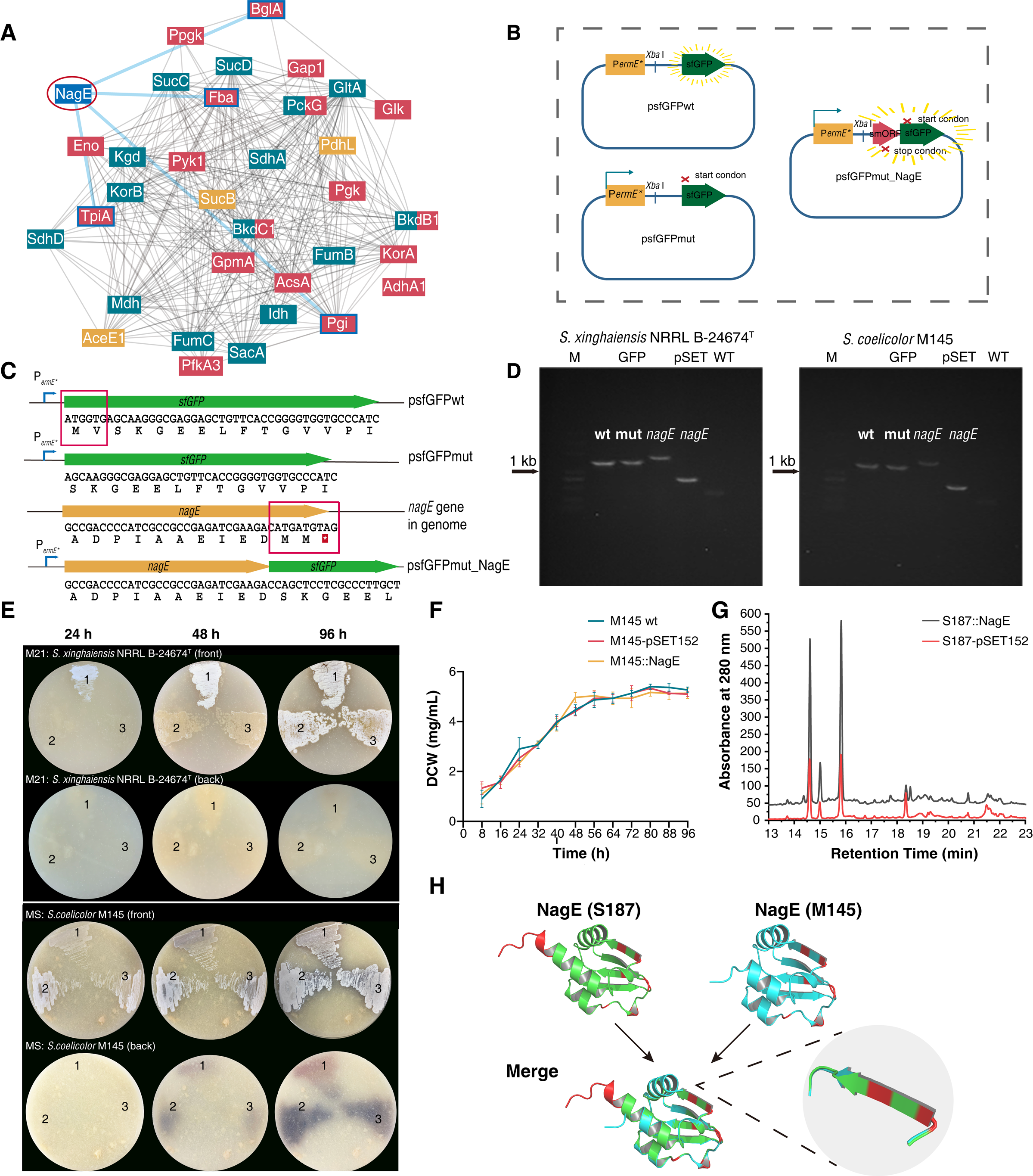

Supplement: Figure S3 — Effect of SEP NagE on growth and metabolism. [file msystems.00245-23-s0003.tif]

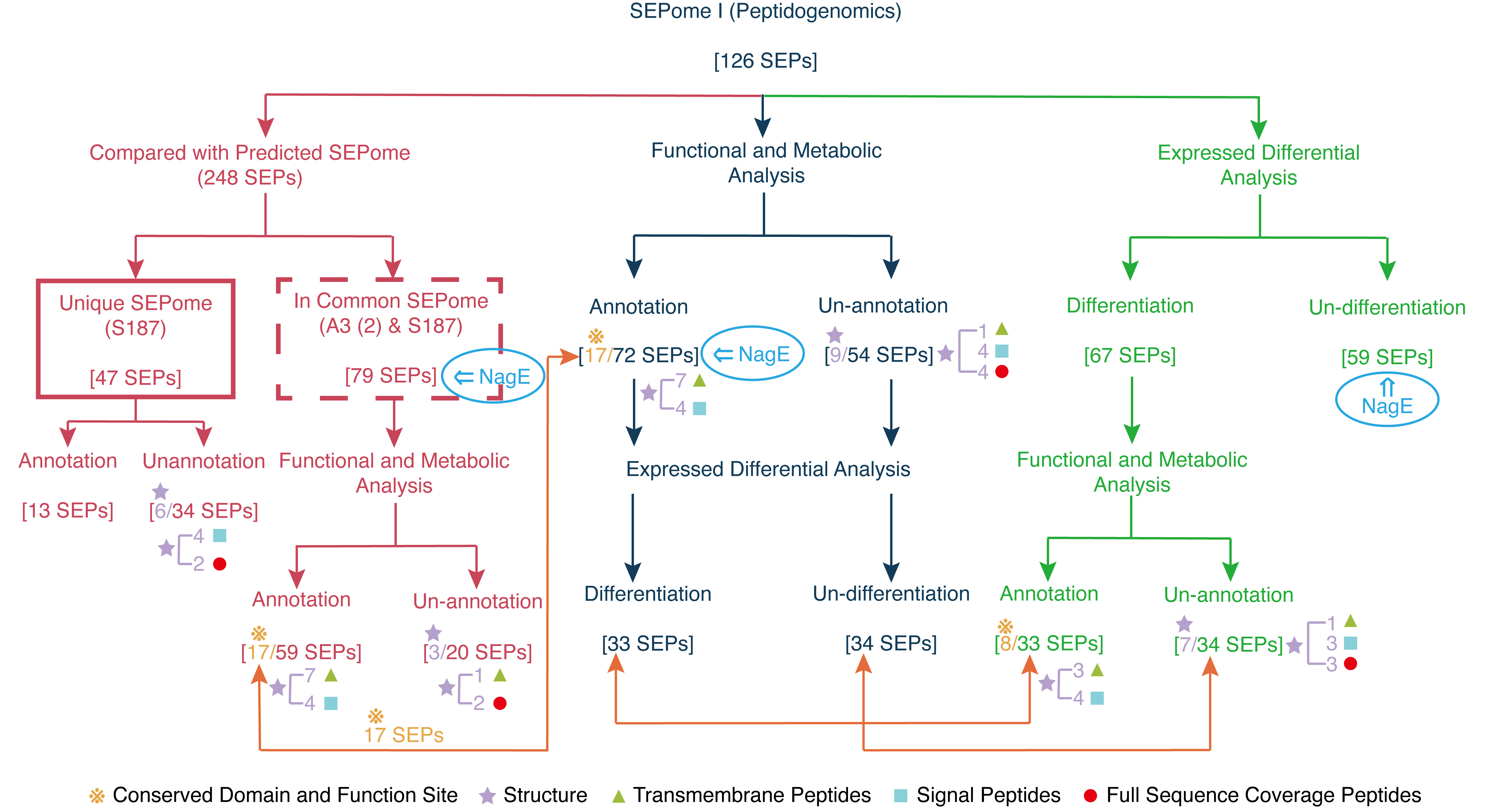

Supplement: Figure S4 — Mining multidimensional analysis of metabolic-related SEPs. [file msystems.00245-23-s0004.tif]
