# Supplementary material for: Multi-integrated approach for unraveling small open reading frames potentially associated with secondary metabolism in Streptomyces
Source: mSystems. 2023 Sep 15;8(5):e00245-23. doi: 10.1128/msystems.00245-23 (PMC10654065; doi:10.1128/msystems.00245-23)
Supplement: Supplemental Material — Legends for Figures S1 to S4; Tables S1 to S9. [file msystems.00245-23-s0005.docx]

# Supplementary materials

**Multi-Integrated** **Approach for Unravelling Small Open Reading Frames Potentially Associated with** **Secondary Metabolism in *Streptomyces***

Si-Min Fan^1, #^, Ze-Qi Li^1, #^, Shi-Zhe Zhang^1^, Liang-Yu Chen^3^, Xi-ying Wei^1^, Jian Liang^4,1,^ Xin-Qing Zhao^2,^ *, Chun Su^1,^ *

^1^National Engineering Laboratory for Resource Developing of Endangered Chinese Crude Drugs in Northwest China, College of Life Sciences, Shaanxi Normal University, China.

^2^State Key Laboratory of Microbial Metabolism, School of Life Sciences and Biotechnology, Shanghai Jiao Tong University, China.

^3^ProteinT (Tianjin) biotechnology Co. Ltd., Tianjin, China.

^4^College of Biology and Geography, Yili Normal University, Yining, China.

#These authors contributed equally to this work.

*Corresponding authors, Emails: [suchun@snnu.edu.cn](mailto:suchun@snnu.edu.cn); xqzhao@sjtu.edu.cn.

# Supplement Tables and Figures

**Table S1. Bacterial strains and plasmids used in this study.**

| **Strain / Plasmid** | **Relevant Phenotype** | **Source** |
| --- | --- | --- |
| *S. xinghaiensis* NRRL B-24674^T^ | Wild type | Laboratory stock |
| *S. coelicolor* M145 | Wild type | Laboratory stock |
| *E. coli* DH5α | F *recA lacZ*M15 | Laboratory stock |
| *E. coli* ET12567/pUZ8002 | *dam*, *dcm*, *hsd*M, *hsd*S, *hsd*R, *cat*R, *tet*R, *tra, neo*, RP4 | Laboratory stock |
| S187 sfGFPwt | Plasmid psfGFPwt in S187; GFP expression | This work |
| S187 sfGFPmut | Plasmid psfGFPmut in S187; no GFP expression | This work |
| S187 sfGFPmut-NagE | Plasimid psfGFPmut-NagE-S187 in S187; GFP expression | This work |
| S187::NagE | The gene *nag*E overexpressed in S187 by strength promoter *kasOp*^*^ | This work |
| S187-pSET152 | S187 with intergrative plasimid pSET152-*kasOp*^*^ | This work |
| M145 sfGFPwt | Plasmid psfGFPwt in S187; GFP expression | This work |
| M145 sfGFPmut | Plasmid psfGFPmut in S187; no GFP expression | This work |
| M145 sfGFPmut-NagE | Plasimid psfGFPmut-NagE-S187 in S187; GFP expression | This work |
| M145::NagE | The *nag*E gene overexpressed in M145 by strength promoter *kasOp*^*^ | This work |
| M145-pSET152 | M145 with integrative plasimid pSET152-*kasOp*^*^ | This work |
| pSET152-*ermEp^*^* | Integrative plasmid containing *ermEp^*^*, ori*T*, att*P*, *ΦC31 int* and *aac(3)IV* | Laboratory stock |
| pSET152-*kasOp*^*^ | Integrative plasmid containing *kasOp^*^*, ori*T*, att*P*, *ΦC31 int* and *aac(3)IV* | Laboratory stock |
| psfGFPwt | *sfGFP* gene without the start codon cloned into pSET152 *ermEp^*^* |  |
| psfGFPmut | *sfGFP* gene without the start codon cloned into pSET152 *ermEp*^*^ | This work |
| psfGFPmut-NagE-S187 | The S187 *nag*E gene without stop codon plasmid cloned into psfGFPmut | This work |
| psfGFPmut-NagE-M145 | The M145 *nag*E gene without stop codon cloned into psfGFPmut | This work |
| pSET152 -NagE-S187 | The S187 *nag*E *gene* over-expression plasmid with strength promoter *kasOp*^*^ | This work |
| pSET152-NagE-M145 | The M145 *nag*E *gene* over-expression plasmid with strength promoter *kasOp*^*^ | This work |

| **Name** | **Sequence (5’-3’)** | **Purpose** |
| --- | --- | --- |
| **Diagnostic PCR and sequencing** | | |
| sfGFPwt-F | TGGTAGGATCTCTAGAATGGTGAGCAAGGGCGAG | Amplifying the complete *sfGFP* gene |
| sfGFPwt-R | GGGCTGCAGGTCGACTTACTTGTACAGCTCGTCCA |  |
| sfGFPmut-F | TGGTAGGATCTCTAGAGTGAGCAAGGGCGAGGAG | Amplifying the *sfGFP* gene without ATGGTG |
| psfGFPmut-NagE-S187-F | TGGTAGGATCTCTAGAGTGCCGAGAACACCAGGAGT | Amplifying the S187 *nag*E gene without stop codon |
| psfGFPmut-NagE-S187-R | CTCGCCCTTGCTCACCATCATGTCTTCGATCTCGGC |  |
| psfGFPmut-NagE-M145-F | CTCCTCGCCCTTGCTGTCTTCGATCTCCGCCGC | Amplifying the M145 *nag*E gene without stop codon |
| psfGFPmut-NagE-M145-R | TGGTAGGATCTCTAGAATGGCCAGCAAGGCTGAGA |  |
| pSET152-NagE-S187-F | GCAGGTCGACTCTAGCTACATCATGTCTTCGATCTC | Amplifying the S187 *nag*E gene |
| pSET152-NagE-S187-R | ACTGGGGGAGTTATGGTGCCGAGAACACCAGGAG |  |
| pSET152-NagE-M145-F | ACTGGGGGAGTTATGATGGCCAGCAAGGCTGAG | Amplifying the M145 *nag*E gene |
| pSET152-NagE-M145-R | GCAGGTCGACTCTAGTCACATCATGTCTTCGATCT |  |
| **Gene-specific primers for RT-qPCR** | | |
| nagE qPCR-F | GACAACATCGAGGAGGTCGA | Amplifying the *nag*E gene |
| nagE qPCR-R | CTGCCCATCTTGACGACGC |  |
| hrdB qPCR-F | ACGTTCTTCCACTGGGTTGG | Amplifying the *hrd*B gene |
| hrdB qPCR-R | GCCGAATCCGAGTCTGTCAT |  |
| **PCR for mutants check** | | |
| ermE^*^ check-F | CGCAACTGTTGGGAAGGG | Check all the above mutants |
| ermE^*^ check-R | TTCCCGACTGGAAAGCGG |  |

**Table S2. Oligonucleotides used in this study.**

**Table S3. Annotated SEPs with conserved structural domains and functional sites in *S. coelicolor* A3 (2) and *S. xinghaiensis* NRRL B-24674^T^.**

| **Function Classification** | **Predicted SEPs (A3 (2) & S187)^27^** | |  | **SEPs from Peptidogenomics (S187)^17^** | |
| --- | --- | --- | --- | --- | --- |
|  | **SCO ID (aa)** | **SEPs Description** |  | **ID (aa)** | **Sig (120_48h)** |
| Ribosome | SCO5736 (95) | 30S ribosomal protein S15 | | RS07760:0:287 (95) | -1 |
|  | SCO4720 (60) | 50S ribosomal protein L30 | | RS12425:0:182 (60) | -1 |
|  | SCO4710 (74) | 50S ribosomal protein L29 | | RS12480:0:224 (74) | -1 |
|  | SCO3906 (96) | 30S ribosomal protein S6 | | RS15070:0:290 (96) | -1 |
|  | SCO4725 (73) | translation initiation factor IF-1 | | RS12400:0:221 (73) | 0 |
| Stress Response | **SCO0527 (67),**  **SCO3731 (67),**  **SCO3748 (67),**  **SCO4295 (67),**  **SCO4505 (67),**  **SCO4684 (67),**  **SCO5921 (67)** | **cold-shock protein (CSP)** | | **RS08820:0:203 (67, 1),**  **RS13025:0:203 (67, 0),**  **RS17250:0:203 (67, 0), RS18780:0:203 (67, 0)** | |
|  | SCO0620 (92) | metal-sensitive transcriptional regulator | | RS29365:0:266 (88) | (-1) |
|  | SCO5187 (80) | glutaredoxin-like protein (GLP) | | RS10085:0:251 (83) | 0 |
| Biosynthetic Process | SCO2911 (92),  SCO4294 (91) | MoaD/ThiS family protein | | RS17245:0:275 (91,1) | |
|  | SCO2108 (66) | sulfur carrier protein ThiS | | RS24050:0:224 (74) | (1) |
|  | SCO4161 (84) | MoaD/ThiS family protein | | No | |
|  | **SCO5841 (93)** | **HPr family phosphocarrier protein (PtsH)** | | **RS07360:0:281 (93)** | **0** |
|  | **SCO2905 (77)** | **phosphoenolpyruvate-dependent sugar PTS (NagE)** | | **RS19465:0:257 (85)** | **0** |
|  | SCO1439 (90) | phosphoribosyl-ATP diphosphatase | | No | |
|  | SCO4682 (69) | 4-oxalocrotonate tautomerase family protein | | No | |
| Nucleic Acid Binding | SCO0148 (81) | helix-turn-helix transcriptional regulator | | No | |
|  | SCO5607 (64) | winged helix-turn-helix domain-containing protein | | No | |
|  | SCO6410 (83) | helix-turn-helix domain-containing protein | | No | |
|  | SCO5592 (79) | RNA-binding protein | | RS08200:0:239 (79) | -1 |
|  | SCO2950 (93) | HU family DNA-binding protein | | RS19300:0:281 (93) | -1 |

**Table S4. Functional SEPs annotated by InterProScan.**

| **SEP ID (aa)** | **InterPro Description** | **Ident/Cov (%)** | **Sig (120_48h)** |
| --- | --- | --- | --- |
| RS07215:0:260 (86) | ribbon-helix-helix protein, CopG | 96.47/98 | -1 |
| RS15970:0:239 (79) | ribbon-helix-helix protein, CopG | 100.00/100 | 0 |
| RS02740:0:158 (52) | arc-type ribbon-helix-helix | 90.38/100 | 0 |
| RS25180:0:221 (73) | helix-turn-helix domain, Actinobacteria-type | 100.00/79 | -1 |
| RS08350:0:233 (77) | transcription regulator AsnC/Lrp, ligand binding domain | 98.70/100 | 0 |
| RS26715:0:293 (97) | transcription factor zinc-finger | 100.00/100 | 0 |
| RS02880:0:293 (97) | DNA-binding protein | 100.00/100 | -1 |
| RS14435:0:296 (98) | thiamine-binding protein | 97.96/100 | 0 |
| RS05060:0:287 (95) | Phosphopantetheine (Ppant) binding ACP domain | 81.69/74 | (1) |
| RS13065:0:287 (95) | integral membrane protein （IMP） | 98.70/81 | 0 |
| RS16880:0:287 (95) | carbohydrate-binding-like fold (CB-like fold) | 100.00/100 | 1 |
| RS07795:0:299 (99) | type VII secretion system ESAT-6-like | 98.99/100 | 0 |
| RS03375:0:275 (91) | type II toxin-antitoxin system, antitoxin Phd/YefM | 93.41/100 | 1 |
| RS06585:0:260 (86) | type II toxin-antitoxin system, antitoxin Phd/YefM | 98.84/100 | 0 |
| RS22200:0:242 (80) | type II toxin-antitoxin system, antitoxin Phd/YefM | 100.00/100 | (-1) |
| RS27865:0:278 (92) | YefM-like superfamily | 100.00/100 | -1 |
| RS17010:0:215 (71) | bacterial antitoxin of type II TA system, VapB | 94.37/100 | (-1) |
| RS13255:0:275 (91) | glutaredoxin-like | 100.00/100 | (-1) |
| RS10505:0:176 (58) | sporulation-like domain | 98.28/100 | 0 |
| RS26490:0:203 (67) | cell growth inhibitor/plasmid maintenance toxic component | 100.00/100 | -1 |

**Table S5. SEPs with a signal peptide and/or transmembrane structures in SEPome I.**

| **SEPs (aa)** | **Annotation Source** | **Proposed Function** | **Ide/Cov (%)** | **S (type)** | **T** | **Sig**  **(120_48h)** | **Class** |
| --- | --- | --- | --- | --- | --- | --- | --- |
| RS15250:65:250 (62) | GO | integral membrane protein (IMP) | 87.93/93 | Sec/SPI | Y | -1 | Com |
| RS13065:0:287 (95) | InterProScan | integral membrane protein (IMP) | 64.94/81 | N | Y | 0 | Com |
| RS14690:0:197 (65) | GO | integral component of membrane | 98.46/100 | N | Y | 0 | Com |
| RS26335:0:299 (99) | GO & KEGG | Sec-independent protein translocase protein TatA/B/E | 100.00/100 | Sec/SPI | Y | 1 | Com |
| RS10520:0:263 (87) | GO & KEGG | Sec-independent protein translocase protein TatA/B/E | 96.55/100 | N | Y | -1 | Com |
| RS12615:0:293 (97) | GO & KEGG | preprotein translocase subunit SecE | 98.97/100 | N | Y | 0 | Com |
| RS11290:0:206 (68) | GO | acyl-CoA carboxylase epsilon subunit (ACC) | 95.59/100 | Tat/SPI | N | 1 | Com |
| RS18945:0:230 (76) | GO | Trm112-like protein | 96.00/98 | Sec/SPII | N | -1 | Com |
| RS09245:0:236 (78) | GO & KEGG | ATP synthase, F0 complex, subunit C | 97.44/100 | N | Y | 0 | Com |
| RS11095:0:296 (98) | - |  |  | N | Y | -1 | Com |
| RS02525:939:661 (92) | - |  |  | Tat/SPI | N | 1 | Uni |
| RS08125:861:631 (76) | - |  |  | Tat/SPI | N | 0 | Uni |
| RS10605:0:230 (76) | - |  |  | Sec/SPI | N | -1 | Uni |
| RS11630:1356:1141 (71) | - |  |  | Tat/SPI | N | -1 | Uni |

“-”: Un-annotation; S: signal peptide site; T: transmembrane structure; Sec/SPI: "standard" secretory signal peptides transported by the Sec translocon and cleaved by Signal Peptidase I (Lep); Sec/SPII: lipoprotein signal peptides transported by the Sec translocon and cleaved by Signal Peptidase II (Lsp); Tat/SPI: Tat signal peptides transported by the Tat translocon and cleaved by Signal Peptidase I (Lep); Y: With conserved structural domain and function site; N: Without conserved structural domain and function site; Com: in common SEPs of both S187 and *S. ceolicolor* A3 (2) strain; Uni: unique SEPs in S187 strain.

**Table S6. Novel SEPs from “*de novo* only” sequencing in *S. xinghaiensis* NRRL B-24674^T^.**

| **Peptide Sequence (aa)** | **SEP Location on Genome**  **(smORF Start-End)** | **Peptide Location**  **on SEP** | **Canonical Protein Location**  **on Genome (ORF Start-End, aa)** | **SEP Type (aa)** |
| --- | --- | --- | --- | --- |
| **Method 1 (1 SEP)** |  |  |  |  |
| F216964: VGA**GPGEGDDAVRPG** (15) | 6, 293, 492--6, 293, 566 | 0/-10 | 6, 291, 884--6, 293, 566 (560) | ISEP (24) |
| F111021: **GPGEGDDAVRPG** (12) | 6, 293, 492--6, 293, 566 | +3/-10 |  |  |
| **Method 2 (3 SEPs)** |  |  |  |  |
| F541327: MRFPGGGE (8) | 908, 104--907, 805 | 0/-92 | 908, 053--907, 805 (82) | ISEP (99) |
| F131566: DDHMTGGELKPL (12) | 4, 101, 210--4, 101, 052 | +17/-24 | 4, 101, 210--4, 101, 052 (52) | ISEP (52)* |
| F218712: SAMSASPVR (9) | 1, 568, 553--1, 568, 341 | +36/-26 | - | CSEP (70) |
| **Method 3 (11 SEPs)** |  |  |  |  |
| F138144: NVFDVVR (7) | 6, 278, 394--6, 278, 426 | +3/-1 | 6, 277, 854--6, 278, 426 (190) | ISEP (10) |
| F324727: MAPVSKL (7) | 1, 589, 112--1, 589, 150 | +0/-6 | 1, 588, 725--1, 589, 150 (141) | ISEP (12) |
| F429113: TFVGGKL (7) | 5, 815, 508--5, 815, 537 | +2/-1 | 5, 814, 533--5, 815, 537 (334) | ISEP (9) |
| F642880: VFGYPARPL (9) | 6, 002, 589--6, 002, 750 | +9/-36 | 6, 001, 881--6, 002, 750 (289) | ISEP (53) |
| F143777: PLELGPAK (8) | 3, 916, 270--3, 916, 241 | +1/-1 | 3, 917, 080--3, 916, 241 (279) | ISEP (9) |
| F211958: VNDPPDVK (8) | 2, 436, 783--2, 436, 532 | +0/-76 | 2, 437, 170--2, 436, 532 (212) | ISEP (83) |
| F451266: EVTTFVGGKL (10) | 5, 815, 502--5, 815, 537 | +1/-1 | 5, 814, 533--5, 815, 537 (334) | ISEP (11) |
| F321848: DPVEELR (7) | 5, 236, 235--5, 236, 489 | +16/-62 | - | CSEP (84) |
| F620858: YVVADLR (7) | 1, 576, 630--1, 576, 406 | +16/-52 | - | CSEP (74) |
| F326490: NGVVELR (7) | 4, 953, 185--4, 952, 883 | +4/-90 | - | CSEP (100) |
| F414413: VLDPDSSPGV (10) | 6, 574, 747--6, 574, 881 | +17/-18 | - | CSEP (44) |

Bold characters: multiple identifications for the same peptide sequences. underline: the sites where MS_2_ spectrum mismatch with annotated peptide sequence. *: common SEP in the result of DB search and *de novo* sequencing.

**Table S7. Thirteen uniquely annotated SEPs of** ***S. xinghaiensis* NRRL B-24674^T^ absent in *S. coelicolor* A3 (2).**

| **Function Classification** | **SEP ID (aa)** | **Proposed Function** | **Ide/Cov (%)** | **Sig (120_48h)** |
| --- | --- | --- | --- | --- |
| Nucleic Acid Binding | RS07215:0:260 (86) | ribbon-helix-helix protein, CopG | 96.47/98 | -1 |
|  | RS15970:0:239 (79) | ribbon-helix-helix protein, CopG | 100.00/100 | 0 |
|  | RS02740:0:158 (52) | arc-type ribbon-helix-helix | 90.38/100 | 0 |
|  | RS02880:0:293 (97) | DNA-binding protein | 100.00/100 | -1 |
| Biosynthetic Process | RS04090:0:254 (84) | (2Fe-2S)-binding protein | 98.81/100 | 0 |
|  | RS28350:1:231 (76) | cytochrome P450 | 98.68/100 | 0 |
|  | RS05060:0:287 (95) | phosphopantetheine (Ppant) binding ACP domain | 81.69/74 | (1) |
| Bacterial Secretion System | RS07795:0:299 (99) | type VII secretion system ESAT-6-like | 98.99/100 | 0 |
| Stress Response | RS17010:0:215 (71) | bacterial antitoxin of type II TA system, VapB | 94.37/100 | (-1) |
|  | RS22200:0:242 (80) | type II toxin-antitoxin system, antitoxin Phd/YefM | 100.00/100 | (-1) |
|  | RS27865:0:278 (92) | YefM-like superfamily | 97.83/100 | -1 |
|  | RS29415:0:221 (73) | CarD family transcriptional regulator | 65.75/100 | 0 |
|  | RS13255:0:275 (91) | glutaredoxin-like | 100.00/100 | (-1) |

**Table S8. The SEPs on the secondary metabolites BGC of *S. xinghaiensis* NRRL B-24674^T^.**

| **BGC Location** | **BGC type** | **Biological activity** | **SEP ID** | **SEP Function** |
| --- | --- | --- | --- | --- |
| Region 1 | citrulassin (RiPP) | antibacterial activity | RS00355:0:215 | 3Fe-4S ferredoxin |
| Region 2 | xinghaimycin (NRPS) | antimicrobial antibiotic | RS02425:0:227 | MbtH-like protein (MLP) |
| Region 4 | largimiycin (NRPS-PKS) | antitumor activity | RS03375:0:275 | type II toxin-antitoxin system,  antitoxin Phd/YefM (Phd/YefM) |
| Region 6 | neomycin  (aminoglycoside) | antimicrobial antibiotic | RS03865:0:248 | Novel SEP |
|  |  |  | RS03850:300:1 | Novel SEP |
|  |  |  | RS03870:542:670 | Novel SEP |
|  |  |  | F541327MRFPGGGE | Novel SEP |
| Region 10 | NRPS | anti-gram-positive bacteria activity | RS05060:0:287 | phosphopantetheine binding ACP domain (Ppant binding ACP) |
| Region 14 | lanthipeptide-class-iii | biocatalyst | RS09155:0:242 | acyl carrier protein (ACP) |
| Region 16 | LL-D49194α1 (T2PKS) | antitumor activity | RS20065:0:287 | phosphopantetheine binding ACP domain (Ppant binding ACP) |
| - | PKS | unknown | RS22495:0:248 | acyl carrier protein (ACP) |
| - | xiamycin (Terpene) | anti-HIV activity | RS20625:0:215 | iron-sulfur cluster binding |

**Table S9. Genome location of smORFs in the xinghaimycin BGC in *S. xinghaiensis* NRRL B-24674^T^.**

| **smORF Location** | **Flanking Gene NCBI Annotation (upstream/downstream)** | **smORF Number (start codon, size of SEPs (aa))** |
| --- | --- | --- |
| 506,769..509,157 | uracil-DNA glycosylase/ATPase | 1(GUG, 50), 2(CUG, 47), 3(GUG, 30) |
| 509,264..511,921 | hypothetical protein/FUSC family protein | 4(AUG, 43), 5(CUG, 26) |
| 510,422..513,343 | FUSC family protein/  PPOX class F420-dependent oxidoreductase | 6(GUG, 31), 7(UUG, 35), 8(UUG, 43), 9(GUG, 45), 10(CUG, 42), 11(UUG, 44), 12(GUG, 49), 13(GUG, 48), 14(CUG, 50), 15(AUG, 43), 16(AUG, 35), 17(CUG, 43), 18(CUG, 31) |
| 512,693..514,974 | PPOX class F420-dependent oxidoreductase/  ABC transporter permease | 19(AUG, 23), 20(CUG, 29), 21(GUG, 49) |
| 517,159..518,917 | hypothetical protein/prephenate dehydrogenase | 22(GUG, 24), 23(GUG, 30), 24(GUG, 41) |
| 518,914..521,756 | PLP-dependent aminotransferase family protein/  4-hydroxyphenylpyruvate dioxygenase | 25(AUG, 49), 26(GUG, 45), 27(CUG, 43), 28(GUG, 23), 29(AUG, 49) |
| 528,725..531,833 | tryptophan 7-halogenase/cation: proton antiporter | 30(UUG, 23), 31(CUG, 23) |
| 530,499..532,149 | cation:proton antiporter/MbtH family protein | 32(CUG, 37), 33(GUG, 45) |
| 565,321..565,398 | NRPS/ABC transporter ATP-binding protein | 34(GUG, 25) |
| 565,395..569,010 | ABC transporter ATP-binding protein/  ParB N-terminal domain-containing protein | 35(AUG, 43), 36(UUG, 32), 37(AUG, 46), 38(CUG, 49), 39(AUG, 44), 40(CUG, 25), 41(CUG, 38), 42(GUG, 42), 43(UUG, 40), 44(AUG, 40), 45(UUG, 34), 46(UUG, 30), |
| 568,015..570,380 | ParB N-terminal domain-containing protein/no annotation | 47(GUG, 31), 48(GUG, 41) |
| 569,460..573,388 | no annotation/LuxR family transcriptional regulator | 49(CUG, 24), 50(UUG, 41) |
| 570,542..574,622 | LuxR family transcriptional regulator/response regulator transcription factor | 51(GUG, 39), 52(UUG, 42), 53(GUG, 50), 54(GUG, 49), 55(GUG, 30), 56(GUG, 29) |

Underline: 13 SEPs were also identified in the smORF database searched by OrfFinder.

**Figure legends**

**Figure S1. The smORFs, smRNAs, and SEPs analysis in *S. xinghaiensis* NRRL B-24674^T^ (S187) and *S.* *coelicolor* A3 (2)**. (**A**) Comparison of results obtained using two software programs (STAR and Bowtie2) in mapping RNA-seq reads to *S. coelicolor* A3 (2) public transcriptomes datasets (SRR13349472, SRR13349473, SRR10011614, SRR10011615, SRR5371191, SRR5371192, and SRR5371193). (**B**) Comparison of four strategies (STAR_BLASTp, STAR_tBLASTn, Bowtie2_BLASTp, and Bowtie2_tBLASTn) in mapping the predicted smORFs of *S. coelicolor* A3 (2) to the S187 genome. (**C**) Genome-predicted smORFs (left) and transcriptome-predicted smRNAs (right) utilize canonical start codons and other start codons to initiate translation. (**D**) Of the 126 SEPs identified, 72 were annotated in database, and 54 un-annotated. (**E**) The presence of two transmembrane helices regions (purple) in membrane proteins RS09245 and RS13065; Identification of signal peptide functionality and cleavage sites within the signal peptide of RS15250. (**F**) The MS_2_ spectrum of the novel identified peptide F111021.

**Figure S2. Analysis of metabolism-related SEPs in** ***S. xinghaiensis* NRRL B-24674^T^ (S187) and *S.* *coelicolor* A3 (2)**. (**A**) The predicted secondary structures of 17 SEPs that contain active and conserved sites in both S187 and *S. coelicolor* A3 (2). (**B**) Cumulative intensity of 464 smRNAs in *S. coelicolor* A3 (2) transcriptome. The top five most abundant smRNAs are labeled in the panel. Among these, the SCO4505 marked in blue is the smRNA coding cold-shock proteins shared with S187 SEPome I. (**C**) RNA in samples of S187 collected at three different fermentation time points (48, 72, and 120 h) is shown on the left. RT-PCR analysis of S187 at three fermentation time points using the primers listed in Supplementary Table 2 on the left side of the illustration. M: DNA molecular ladder (DL 5000). T-RNA and T-cDNA were obtained using RNA and cDNA of the samples as the respective templates. (**D**) BLAST sequence alignment analysis and the predicted secondary structures of SEP MLP in other modal *Streptomyces* species and microorganism. The conserved active sites are marked by stars. (**E**) Through six-frame translation in the S187 genome, 56 smORFs were identified in the xinghaimycin BGC. Of these, *sin*E encodes SEP MLP, which is boxed by red rectangles. (**F**) The protein-protein interaction (PPI) network of xinghaimycin biosynthesis-related proteins in S187 was revealed by functional protein association network (STRING) analysis. (**G**) Volcano plot representing log2 fold change as a function of the p-value for expression of 1,952 proteins compared between S187 samples at 48 and 120 h. Significantly upregulated proteins are represented as red dots, and significantly downregulated proteins as green dots. (**H**) KEGG pathway enrichment analysis of significantly differentially expressed SEPs. The top one differentially expressed proteins are enriched in the ribosomal pathway.

**Figure S3. Effect of SEP NagE on growth and metabolism** **in *S. xinghaiensis* NRRL B-24674^T^ (S187) and *S. coelicolor* A3 (2)**. (**A**) The interaction map of the NagE (blue rectangle), TCA cycle pathway (green rectangle), gluconeogenesis pathway (red rectangle), and pyruvate metabolism pathway (yellow rectangle) related proteins. The protein-protein interaction between NagE and gluconeogenesis pathway-related proteins are marked in blue lines. (**B**) The illustration of construction of fluorescent expression systems in S187 and *S. coelicolor* A3 (2). psfGFPwt: pSET152 *ermE*^*^ contained the full sequence of sfGFP. psfGFPmut: pSET152 *ermE*^*^contained the sequence of sfGFP without stop codons. psfGFPmut-smORF: psfGFPmut contained the sequence of smORF without a stop codon. (**C**) Schematic diagram of the construction of the fluorescent vector, and deleted sequences is boxed using red rectangles. (**D**) PCR analyses of the wild-type strain, the NagE sfGFP-expressing vectors, and NagE overexpression mutants carried out using the primers listed in Supplementary Table 2. M: DNA molecular ladder (DL 5000); GFP (left): using genomic DNA of S187 sfGFPwt, S187 sfGFPmut, S187 sfGFPmut-NagE, and S187::NagE as a template; pSET (left): genomic DNA of S187::NagE as a template; WT (left): using genomic DNA of S187 wt as a template. GFP (right): using the genomic DNA of M145 sfGFPwt, M145 sfGFPmut, M145 sfGFPmut-NagE, and M145::NagE as templates; pSET (right): using the genomic DNA of M145::NagE as a template; WT (right): using the genomic DNA of S187 wt as a template. (**E**) Growth phenotype of S187 and M145 on M21 and MS solid medium after 24, 48, and 96 h. 1: S187/M145::NagE; 2: S187/M145-pSET152; 3: S187/M145 wt. (**F**) Growth curves of wild type and *nag*E over-expression mutant of M145 cultures cultivated in TSB liquid medium. Each point represents the mean ± standard deviation of three independent cultures. (**G**) High-performance liquid chromatography (HPLC) chromatograms demonstrating the changing in secondary metabolic production in *nag*E over-expression mutant. (**H**) The predicted protein structures of SEP NagE in S187 and *S. coelicolor* A3 (2) were predicted using ColabFold. The red mark and enlarged part of the image show differences between the two proteins.

**Figure S4. Mining multidimensional analysis of *S. xinghaiensis* NRRL B-24674^T^ (S187) metabolic-related SEPs**. The mining of metabolism-related SEPs can be divided into three analysis modules: comparison with predicted SEPome (red), functional and metabolic analysis (blue), and expressed differentiation analysis (green). Using the predicted SEPome module, 248 putative SEPs based on the predicted SEPome were compared with 126 SEPs in SEPome I to identify SEPs that might be prevalent in *Streptomyces*. In the functional and metabolic analysis module, the 126 SEPs detected were functionally annotated, and conserved domains, active sites, and secondary structures of SEPs were evaluated to determine the functions of SEPs. Differentially expressed proteins were counted using an expressed differentiation analysis module. The data from the three modules were cross-referenced to identify biologically significant SEPs (functional SEP information is marked in colors and shapes at the bottom of the illustration).
